# A group 3 medulloblastoma stem cell program is maintained by OTX2-mediated alternative splicing

**DOI:** 10.1038/s41556-024-01460-5

**Published:** 2024-07-18

**Authors:** Olivier Saulnier, Jamie Zagozewski, Lisa Liang, Liam D. Hendrikse, Paul Layug, Victor Gordon, Kimberly A. Aldinger, Parthiv Haldipur, Stephanie Borlase, Ludivine Coudière-Morrison, Ting Cai, Emma Martell, Naomi M. Gonzales, Gareth Palidwor, Christopher J. Porter, Stéphane Richard, Tanveer Sharif, Kathleen J. Millen, Brad W. Doble, Michael D. Taylor, Tamra E. Werbowetski-Ogilvie

**Affiliations:** 1 The Arthur and Sonia Labatt Brain Tumour Research Centre, The Hospital for Sick Children, Toronto, Ontario Canada; 2 Developmental and Stem Cell Biology Program, The Hospital for Sick Children, Toronto, Ontario Canada; 3 Genomics and Development of Childhood Cancers, Institut Curie, PSL University, Paris, France; 4 INSERM U830, Cancer, Heterogeneity, Instability and Plasticity, Institut Curie, PSL University, Paris, France; 5 SIREDO Oncology Center, Institut Curie, Paris, France; 6 Department of Biochemistry and Medical Genetics, Rady Faculty of Health Sciences, University of Manitoba, Winnipeg, Manitoba Canada; 7 Department of Medical Biophysics, University of Toronto, Toronto, Ontario Canada; 8 Center for Integrative Brain Research, Seattle Children’s Research Institute, Seattle, WA USA; 9 Department of Pediatrics, Division of Genetic Medicine, University of Washington, Seattle, WA USA; 10 Brotman Baty Institute for Precision Medicine, Seattle, WA USA; 11 Segal Cancer Center, Lady Davis Institute for Medical Research and Gerald Bronfman Department of Oncology, McGill University, Montreal, Quebec Canada; 12 Departments of Biochemistry, Human Genetics and Medicine, McGill University, Montreal, Quebec Canada; 13 Department of Pathology, Rady Faculty of Health Sciences, University of Manitoba, Winnipeg, Manitoba Canada; 14 Department of Human Anatomy and Cell Science, Rady Faculty of Health Sciences, University of Manitoba, Winnipeg, Manitoba Canada; 15 Texas Children’s Hospital, Houston, TX USA; 16 Department of Pediatrics, Hematology/Oncology, Baylor College of Medicine, Houston, TX USA; 17 Ottawa Bioinformatics Core Facility, Ottawa Hospital Research Institute, Ottawa, Ontario Canada; 18 Department of Pediatrics and Child Health, Rady Faculty of Health Sciences, University of Manitoba, Winnipeg, Manitoba Canada; 19 Department of Laboratory Medicine and Pathobiology, University of Toronto, Toronto, Ontario Canada; 20 Department of Surgery, University of Toronto, Toronto, Ontario Canada; 21 Texas Children’s Cancer and Hematology Center, Houston, TX USA; 22 Department of Neurosurgery, Baylor College of Medicine, Houston, TX USA; 23 Department of Neurosurgery, Texas Children’s Hospital, Houston, TX USA; 24 Dan L Duncan Comprehensive Cancer Center, Baylor College of Medicine, Houston, TX USA

**Keywords:** Cancer stem cells, Differentiation, CNS cancer

## Abstract

OTX2 is a transcription factor and known driver in medulloblastoma (MB), where it is amplified in a subset of tumours and overexpressed in most cases of group 3 and group 4 MB. Here we demonstrate a noncanonical role for OTX2 in group 3 MB alternative splicing. OTX2 associates with the large assembly of splicing regulators complex through protein–protein interactions and regulates a stem cell splicing program. OTX2 can directly or indirectly bind RNA and this may be partially independent of its DNA regulatory functions. OTX2 controls a pro-tumorigenic splicing program that is mirrored in human cerebellar rhombic lip origins. Among the OTX2-regulated differentially spliced genes, *PPHLN1* is expressed in the most primitive rhombic lip stem cells, and targeting *PPHLN1* splicing reduces tumour growth and enhances survival in vivo. These findings identify OTX2-mediated alternative splicing as a major determinant of cell fate decisions that drive group 3 MB progression.

## Abstract (web summary)

Werbowetski-Ogilvie, Taylor and colleagues report a noncanonical role for OTX2 in regulating alternative splicing and controlling a stem cell and pro-tumorigenic splicing program in group 3 medulloblastoma.

## Main

Medulloblastoma (MB) is a heterogeneous malignant paediatric brain tumour that is currently divided into four molecular subgroups: WNT, sonic hedgehog (SHH), group 3 and group 4 (ref. ^1^). Group 3 MB tumours account for 25% of MB cases, are highly metastatic and exhibit the worst prognosis, with infants displaying 5- and 10-year overall survival rates of 45 and 39%, respectively^2^. They are very aggressive and the mechanisms regulating cell fate decisions are poorly understood. However, recent studies have identified the precise cell of origin—one that is unique to the human cerebellum^3,4^. Group 3 MBs mostly arise from defects in progenitor cells from the human cerebellar rhombic lip subventricular zone (RL^SVZ^), whereas the more deadly group 3γ MBs^5^ display enrichment for the primitive rhombic lip ventricular zone (RL^VZ^) that primarily comprises stem cells^6^. Cancer cells that exhibit aberrant and persistent stem/progenitor cell signatures are associated with tumour propagation, metastasis and drug resistance^7^. Thus, further characterization and targeting of the most primitive MB cells that display these signatures will lead to the development of new treatments that have fewer toxic effects on the nervous systems of young patients.

Amplification or overexpression of the gene transcribing the neurodevelopmental transcription factor orthodenticle homeobox 2 (OTX2) is a molecular hallmark of group 3 MB. Over 80% of group 3 MB tumours exhibit either recurrent gain or overexpression of this homeobox gene^8^. To date, OTX2 studies in group 3 MB have primarily focused on its role in promoting tumour growth through the activation of cell cycle genes^9–11^, as well as the association of OTX2 with active enhancer elements to modulate the chromatin landscape^12^. We, and others, recently demonstrated that OTX2 is a master regulator of group 3 (and group 4) MB cell fate decisions that favour enhanced self-renewal and the retention of a primitive cerebellar rhombic lip signature at the expense of differentiation^3,4,13,14^. OTX2 broadly restricts the expression of neurodevelopmental transcription factors resulting in downstream effects on pathways controlling protein synthesis, such as mammalian target of rapamycin complex 1 (mTORC1), that regulate cell fate^15^. These findings expanded the scope of this multifaceted transcription factor in group 3 MB and suggest that OTX2 may play a more complex role in gene expression beyond transcription.

In this Article, we characterize the group 3 MB alternative splicing landscape and demonstrate an unexpected role for OTX2 in regulating alternative splicing. OTX2 regulates the expression of genes transcribing proteins that make up the large assembly of splicing regulators (LASR) protein complex. Furthermore, it associates with these splicing proteins (either directly or indirectly) and modulates their localization to help stabilize the complex and sustain a group 3 MB stem cell splicing program. OTX2-regulated differentially spliced genes (DSGs) control group 3 MB progression and are associated with the most aggressive MB subtypes. We propose that OTX2-mediated alternative splicing is a key determinant of cell fate decisions favouring maintenance of the stem cell state that ultimately drives group 3 MB progression.

## Results

### OTX2 associates with splicing regulators

**Fig. 1:**
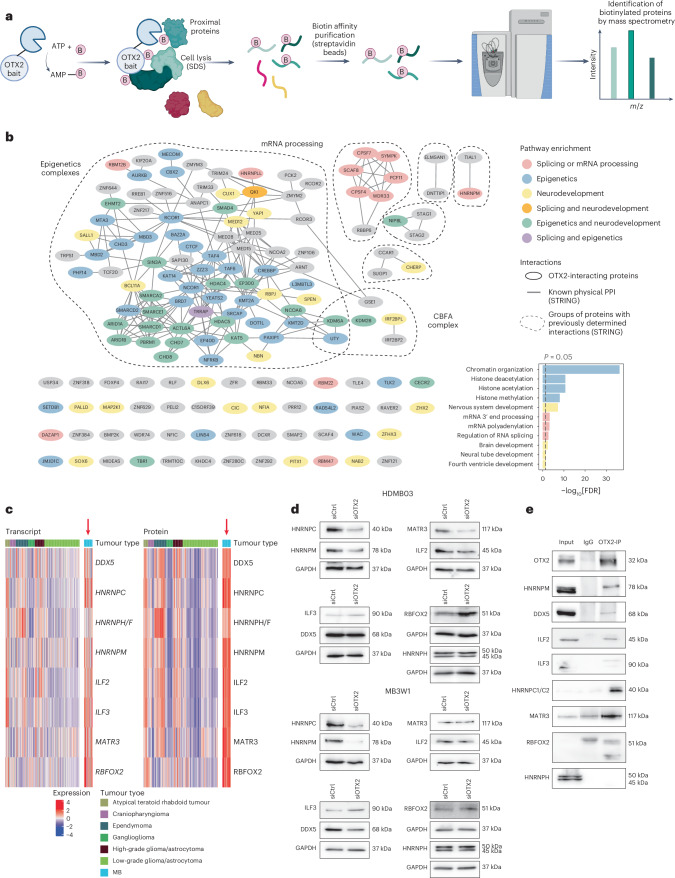
OTX2 associates with core components of the LASR complex. **a**, Schematic depicting the TurboID workflow used to identify OTX2 proximal interacting proteins. **b**, Known PPIs (edges) between OTX2-interacting proteins (nodes), as identified by TurboID experiments. PPIs were determined by STRING analysis, with sub-setting only for experimentally determined physical interactions with a medium (0.400) confidence score or above. The node colours correspond to the enriched pathways displayed in the inset. Pathway significance was assessed using g:Profiler and the GO:BP database with FDR correction. The dotted lines encompass clusters of OTX2-interacting proteins with previously determined interactions. OTX2 was not shown to decrease the complexity. *n* = 4 biological replicates for OTX2-Turbo samples and *n* = 3 biological replicates for control (Ctrl-Turbo) samples. **c**, Heatmap illustrating transcript (left) and transcribed protein (right) levels of genes transcribing members of the LASR complex across 218 samples representing multiple classes of paediatric brain cancer. Individual samples are vertically aligned, with the 22 MB samples coloured blue. Signalling components absent from the ProTrack database have been omitted. **d**, Western blots depicting LASR complex member protein levels following OTX2 silencing in HDMB03 and MB3W1 tumourspheres. GAPDH served as a loading control. *n* = 2 independent experiments per cell line with similar results. **e**, Co-IP validation of endogenous OTX2 protein association with HNRNPM and other components of the LASR complex in HDMB03 tumourspheres. *n* = 5 independent experiments with similar results. B, biotin; CBFA, core binding factor alpha; IgG, immunoglobulin G; SDS, sodium dodecyl sulfate. Panel **a** created with BioRender.com.

**Extended Data Fig. 1:**
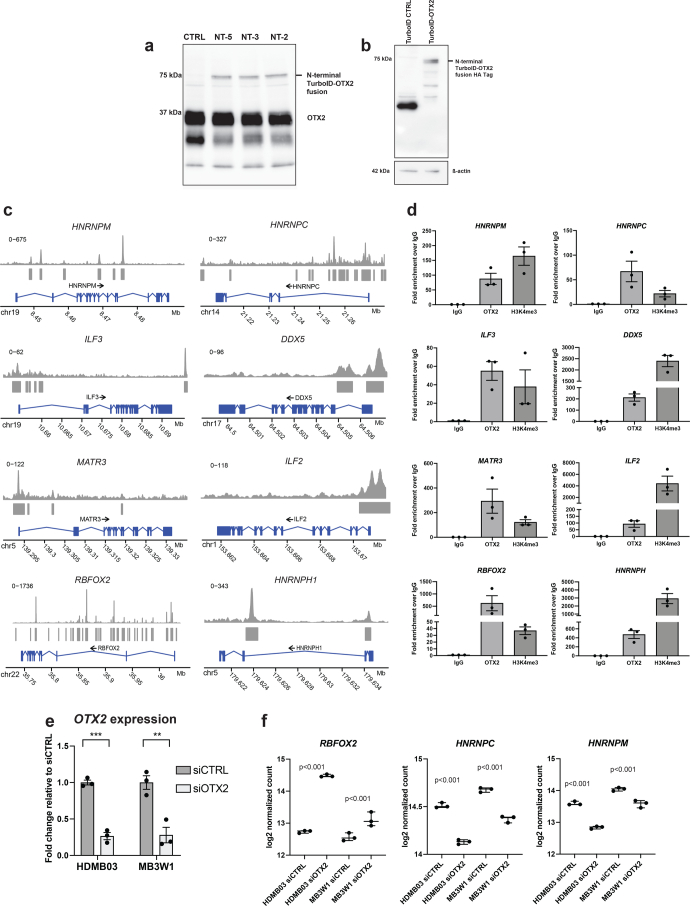
Turbo ID validation and LASR complex gene expression in Group 3 MB tumorspheres following OTX2 silencing. **a**. Immunoblot showing successful expression of the TurboID-OTX2 fusion protein when the TurboID construct is fused to the N-terminal of OTX2. Three independent clones (NT-2, NT-3, and NT-5 were assessed for N-terminal TurboID-OTX2 expression at 75 kDa relative to endogenous levels of OTX2 at 32/37 kDa. N = 3 experiments with similar results. **b**. Immunoblot showing representative TurboID-OTX2 fusion protein relative to TurboID-CTRL in HDMB03 tumorspheres that were utilized for proteomics analyses. β-actin serves as a loading control. N = 4 experiments with similar results. **c**. OTX2 enrichment is observed at regulatory elements on LASR complex gene members. From top to bottom, each sub-panel shows: (1) OTX2 ChIP-seq coverage depth, with the range shown numerically in the top left, (2) OTX2 peaks called by MACS2, (3) exons of the gene depicted in the sub-panel, and (4) chromosome number and chromosomal coordinates in Mbp. Images represent un-normalized read depth. **d**. ChIP-qPCR validation of OTX2 ChIP-sequencing data showing enrichment of OTX2 on all 8 LASR complex genes suggesting that OTX2 can regulate the expression of these complex members. N = 3 biological replicates for each gene. IgG was used as a negative control and H3K4me3 was used as a positive control for enrichment. Error bars: SEM. **e**. qPCR validation of OTX2 silencing in HDMB03 and MB3W1 tumorspheres. Data are normalized relative to scramble controls (siCTRL). Error bars: SEM. p < 0.01**, p < 0.001***. HDMB03, p = 0.0002; MB3W1, p = 0070. N = 3 biological replicates for each condition and cell line. Results were analyzed using an unpaired t-test. **f**. Log2 normalized counts for RBFOX2, HNRNPC and HNRNPM following OTX2 silencing in HDMB03 and MB3W1 tumorspheres obtained from RNA-seq data. Error bars: SEM. N = 3 biological replicates for each condition. p < 0.001 for all conditions. Results were analyzed using a Wald test, with FDR correction. Source data

We previously showed that OTX2 both regulates the expression of and interacts with members of epigenetic protein complexes^15^. To expand these findings and more broadly interrogate the OTX2 protein interactome in group 3 MB, we performed TurboID experiments in group 3 HDMB03 tumourspheres (Fig. 1a and Extended Data Fig. 1a,b). As expected, we identified many epigenetic regulators, in line with the well-documented role for OTX2 in controlling the chromatin landscape^12^. Interactions with the core binding factor alpha complex were also identified, consistent with our previous findings demonstrating an association between OTX2 and this protein complex in regulating cell fate (Fig. 1b and Supplementary Tables 1 and 2)^3^. OTX2 also interacted with T-box brain transcription factor 1, which undergoes a stereotypic somatic point mutation (leading to substitution of glycine for cysteine at position 275 in the transcribed protein (p.Gly275Cys)) specifically in group 4 MB and is mutually exclusive with *OTX2* amplifications^3,16^.

Unexpectedly, we also identified a significant number of OTX2 interactors involved in pre-messenger RNA (pre-mRNA) alternative splicing, such as heterogeneous nuclear ribonucleoprotein M (HNRNPM)—a member of the multimeric LASR complex (Fig. 1b and Supplementary Tables 1 and 2), which includes the well-known RNA-binding protein (RBP) RNA binding fox-1 homolog 2 (RBFOX2) known to regulate splicing^17,18^. Transcriptomic and proteomic analyses of seven types of paediatric brain cancer^19^, including MB, revealed that all LASR complex members are overexpressed in MB at both the transcript and protein level compared with other paediatric brain tumour types (Fig. 1c). Further interrogation of our published chromatin immunoprecipitation followed by sequencing (ChIP-seq) data for OTX2 (refs. ^13,15^) demonstrated that OTX2 also binds to regulatory elements on all genes that transcribe members of the LASR protein complex (Extended Data Fig. 1c) and this was confirmed by ChIP combined with quantitative PCR (ChIP-qPCR; Extended Data Fig. 1d). Western blot analyses for all LASR proteins following OTX2 silencing (Fig. 1d) demonstrated that RBFOX2 is increased, whereas HNRNPM and HNRNPC are decreased, in HDMB03 and MB3W1 tumourspheres. The same patterns were observed at the transcript level when OTX2 was silenced (Extended Data Fig. 1e,f). Strikingly, co-immunoprecipitation (co-IP) experiments supported our TurboID data and demonstrated that endogenous OTX2 interacts—either directly or indirectly—with HNRNPM as well as the majority of LASR complex members (Fig. 1e).

**Extended Data Fig. 2:**
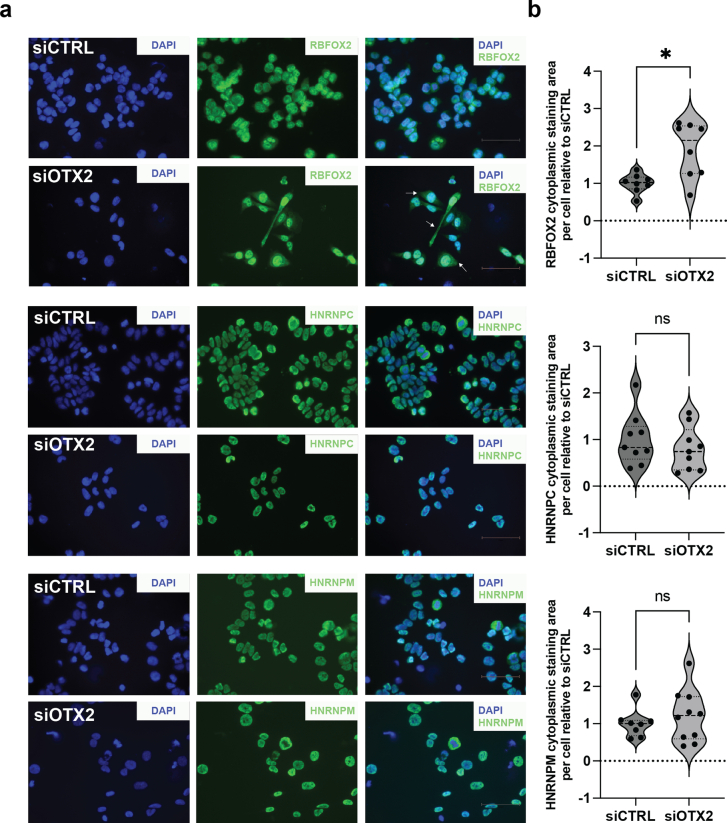
Immunofluorescence demonstrating that RBFOX2, but not HNRNPM or HNRNPC, exhibits an increase in cytoplasmic staining when OTX2 is silenced. **a**. Representative immunofluorescent images depicting localization of RBFOX2, HNRNPC and HNRNPM following OTX2 silencing. Arrows denote RBFOX2 levels in the cytoplasmic extensions of differentiating HDMB03 cells following OTX2 silencing. Blue: DAPI, Green: RBFOX2, HNRNPC or HNRNPM. Scale bar: 75 μm. **b**. Quantification representing 8–10 images for each antibody per condition. For RBFOX2: n = 539 cells for siCTRL and n = 208 cells for the siOTX2 condition were analyzed. For HNRNPM: n = 547 cells for siCTRL and n = 332 cells for the siOTX2 condition were analyzed. For HNRNPC: n = 507 for siCTRL and n = 264 cells for the siOTX2 condition were analyzed. P = 0.01 for RBFOX2. N = 3 independent experiments. Results were analyzed using a Welch’s t-test. Source data

Focusing on RBFOX2, HNRNPC and HNRNPM, we also interrogated the intracellular localization of these RBPs following OTX2 silencing using immunofluorescence. Unexpectedly, RBFOX2, but not HNRNPC or HNRNPM, showed an increase in the cytoplasm when OTX2 was silenced (Extended Data Fig. 2). Typically, cytoplasmic levels of RBFOX2 are low^20,21^; however, studies have shown that RBFOX2 is redistributed to the cytoplasm in motor neurons from experimental models of amyotrophic lateral sclerosis^22^, as well as in cells that undergo epithelial-to-mesenchymal transition and differentiation^23^. These findings suggest that OTX2 not only associates with LASR complex members through protein–protein interactions, but may also stabilize RBFOX2 nuclear localization to regulate alternative splicing.

**Extended Data Fig. 3:**
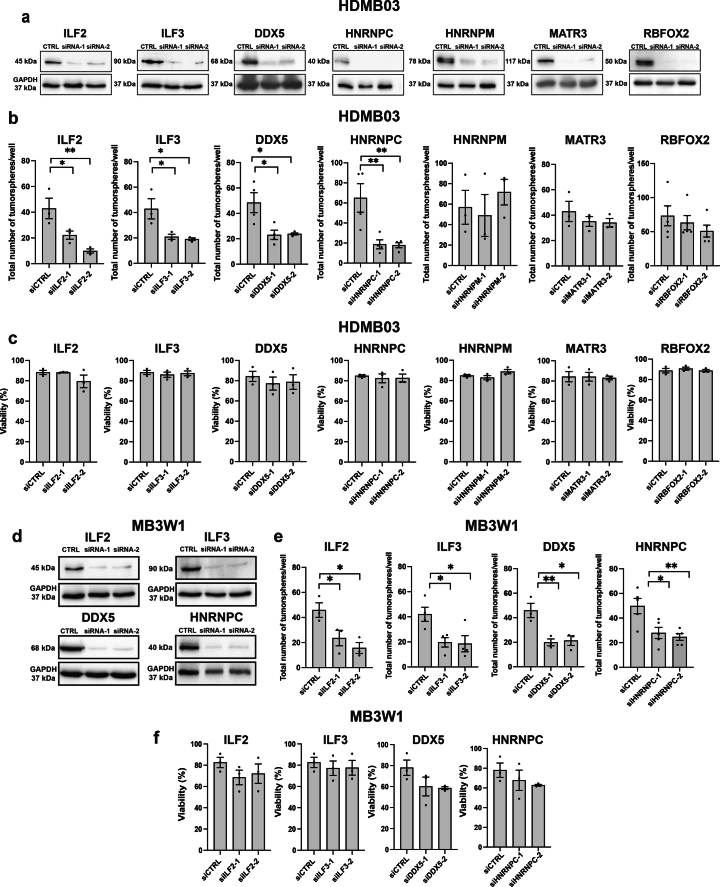
Silencing of LASR complex members decreases tumorigenic properties in Group 3 MB tumorspheres. **a**. Immunoblot validations of individual LASR complex gene silencing following HDMB03 tumorsphere treatment with 2 independent siRNAs for each gene relative to siCTRL. GAPDH serves as a loading control for each blot. **b**. Quantification of HDMB03 total tumorsphere number following 5 day treatment with siCTRL, or siRNAs for LASR complex members. Error bars: SEM. p < 0.05*, p < 0.01**. ILF2 p = 0.0468, p = 0.0064, ILF3 p = 0.0306, p = 0.0217, DDX5 p = 0.0113, p = 0.0133, HNRNPC p = 0.0084, p = 0.0075. N = 3 or 4 biological replicates. Results were analyzed using one-way ANOVA and a Dunnett’s test for multiple comparisons. **c**. Quantification of HDMB03 tumorsphere viability following 72 hours treatment with siCTRL, or siRNAs for LASR complex members. Error bars: SEM. Results were analyzed using one-way ANOVA and a Dunnett’s test for multiple comparisons. **d**. Immunoblot validations of ILF2, ILF3, DDX5 and HNRNPC gene silencing following MB3W1 tumorsphere treatment with 2 independent siRNAs for each gene relative to siCTRL. GAPDH serves as a loading control for each blot. **e**. Quantification of MB3W1 total tumorsphere number following 5 day treatment with siCTRL, or siRNAs for the LASR complex members ILF2, ILF3, DDX5, and HNRNPC. Error bars: SEM. p < 0.05*, p < 0.01**. ILF2 p = 0.0496, p = 0.0144, ILF3 p = 0.0301, p = 0.0241, DDX5 p = 0.0098, p = 0.0127, HNRNPC p = 0.0122, p = 0.005. N = 3–5 biological replicates for each. Results were analyzed using one-way ANOVA and a Dunnett’s test for multiple comparisons. **f**. Quantification of MB3W1 tumorsphere viability following 72 hours treatment with siCTRL, or siRNAs for the LASR complex members ILF2, ILF3, DDX5, and HNRNPC. Error bars: SEM. Results were analyzed using one-way ANOVA and a Dunnett’s test for multiple comparisons. Source data

To test the importance of LASR complex members in group 3 MB progression, we silenced LASR complex proteins that interacted with OTX2 as well as RBFOX2. Knockdown of interleukin enhancer-binding factor 2 (ILF2), ILF3, DEAD box helicase 5 (DDX5) or HNRNPC resulted in a significant decrease in tumoursphere numbers without affecting viability in HDMB03 (Extended Data Fig. 3a–c) and MB3W1 group 3 MB cells (Extended Data Fig. 3d–f), suggesting a potential oncogenic role in regulating group 3 MB stem cell function. We conclude that OTX2 regulates the expression and localization of LASR complex proteins, which it subsequently binds (either directly or indirectly), and that this activity helps to maintain a group 3 MB stem cell program.

### OTX2 silencing results in significant splicing alterations

**Fig. 2:**
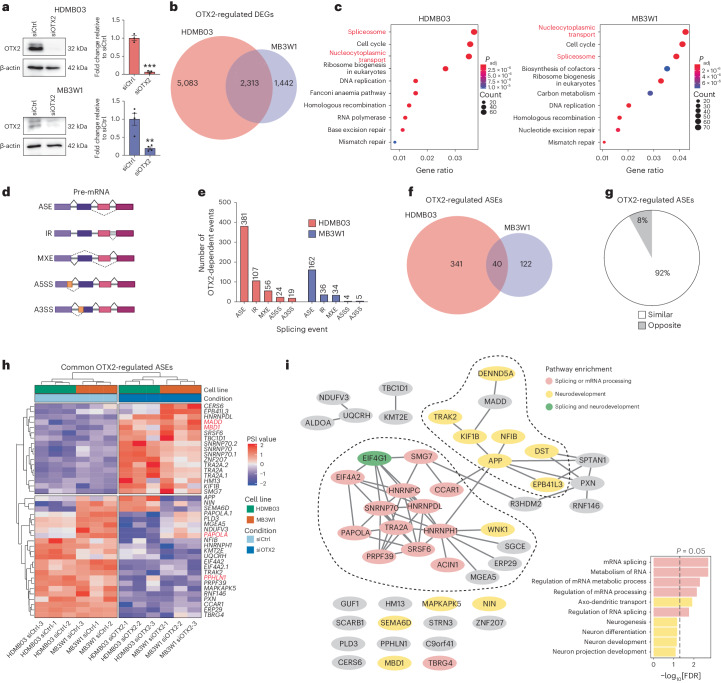
OTX2 silencing results in significant splicing alterations. **a**, Representative western blots (left) and quantification (right) of OTX2 silencing in HDMB03 and MB3W1 tumourspheres. GAPDH served as a loading control. The error bars represent s.e.m. (*n* = 3 (HDMB03) and 4 (MB3W1) biological replicates per condition). Statistical significance was determined by unpaired *t*-test (***P = 0.0002 (HDMB03) and **P = 0.0033 (MB3W1)). **b**, Overlap in DEGs with significant differences in expression following OTX2 silencing in HDMB03 (red) and MB3W1 (blue) tumourspheres. *P* < 10 × 10^−50^ by hypergeometric probability, with an expected overlap of 1,603. The data represent three biological replicates (siCtrl and siOTX2) for tumourspheres from each cell line (FDR < 0.05; fold change > 2). **c**, KEGG pathway analyses on OTX2-downregulated genes. Genes associated with the spliceosome and nucleocytoplasmic transport (red labels) were significantly downregulated following OTX2 silencing in HDMB03 (left) and MB3W1 (right) tumourspheres. Statistical significance was determined by right-tailed Fisher’s exact test. **d**, Schematic depicting the different types of splicing events. **e**, Numbers of OTX2-dependent splicing events identified by rMATS following OTX2 silencing in HDMB03 (red) and MB3W1 (blue) tumourspheres. **f**, Venn diagram depicting overlap specifically in OTX2-regulated ASEs in HDMB03 (red) and MB3W1 (blue) tumourspheres. *P* < 10 × 10^−50^ by hypergeometric probability, with an expected overlap of 2. The data represent *n* = 3 biological replicates (siCtrl and siOTX2) for tumourspheres from each cell line. **g**, Proportion of common target ASEs following OTX2 silencing (as shown in **f**; *n* = 40) that were similar (white) or opposite (grey), depending on whether their change in PSI value varied in the same or opposite direction, respectively. **h**, Heatmap of normalized PSI values from OTX2-regulated ASEs common to both HDMB03 and MB3W1 siCtrl and siOTX2 tumourspheres. ASEs highlighted in red were selected for further validation. **i**, Interaction map showing PPIs (edges) between protein products (nodes) of 48 common OTX2-regulated DSGs. PPIs were determined using STRING analysis, with sub-setting for only experimentally determined interactions with a low (0.150) confidence score or above. Pathway significance was assessed with g:Profiler using the GO:BP and Reactome databases with FDR correction. A3SS, alternative 3′ splice site; A5SS, alternative 5′ splice site; IR, intron retention; MXE, mutually exclusive exon; *P*_adj_, adjusted *P* value. Source data

**Extended Data Fig. 4:**
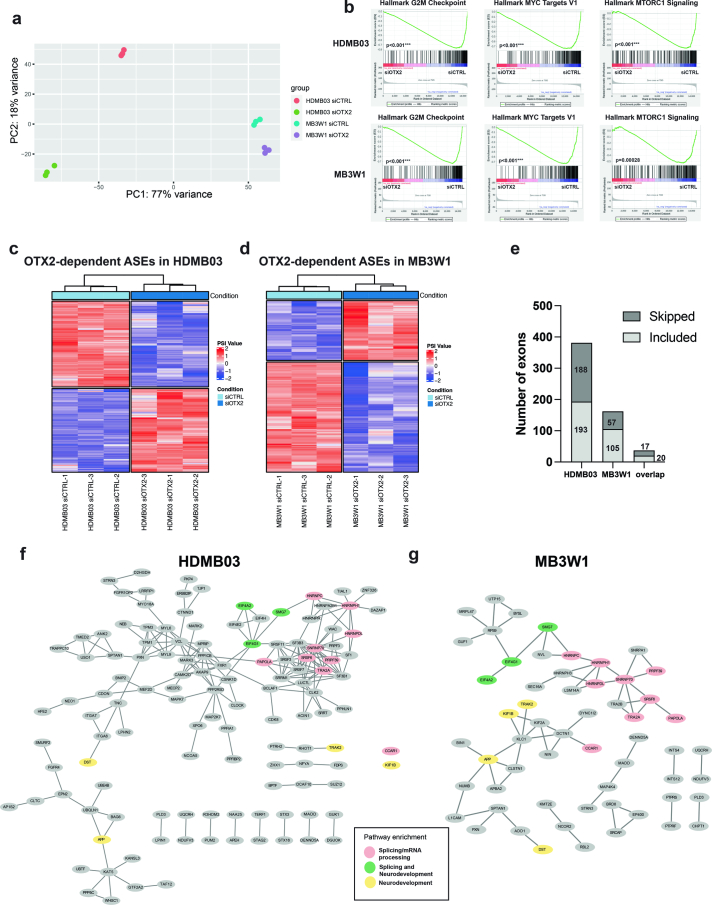
OTX2 silencing is associated with significant splicing alterations in HDMB03 and MB3W1 Group 3 MB tumorspheres. **a**. Principal component analyses (PCA) demonstrating clustering of replicates for siCTRL and siOTX2 tumorspheres derived from HDMB03 and MB3W1 cells. **b**. GSEA demonstrating that hallmark genes associated with G2M checkpoint, MYC targets V1 and MTORC1 signaling are enriched in genes sets that are downregulated in siOTX2 HDMB03 (upper) and MB3W1 (lower) tumorspheres. padj < 0.05* for all signatures. **c-d**. Heat map of normalized PSI values from OTX2-regulated alternatively spliced exons (ASEs) in HDMB03 (c) or MB3W1 (d) siCTRL and siOTX2 tumorspheres. **e**. Bar graph depiction of the number of OTX2-regulated alternatively spliced exons (ASEs) in HDMB03 and MB3W1 tumorspheres that are skipped (dark grey) or included (light grey). **f-g**. Interaction maps showing protein-protein interactions (PPIs) (edges) between protein products (nodes) of OTX2-regulated significantly differentially spliced genes (DSGs) in HDMB03 (f) and MB3W1 (g) tumorspheres. PPIs were determined using STRING analysis, sub-setting for only experimentally determined interactions with a medium (0.4) confidence score or above. Node colours correspond to DSGs associated with the indicated pathways that overlap between the 2 cell lines.

To further examine a potential role for OTX2 in controlling splicing, we performed bulk RNA sequencing (RNA-seq) on stem cell-enriched tumourspheres derived from two group 3 MB cell lines after OTX2 silencing (Fig. 2a and Extended Data Fig. 4a)^3^. The transcriptomic profiles of biological replicates were highly similar (Extended Data Fig. 4a and Supplementary Tables 3 and 4), with 2,313 differentially expressed genes (DEGs) in common (Fig. 2b). Among the 2,313 shared DEGs in HDMB03 and MB3W1 tumourspheres, 1,573 genes had higher expression and 740 genes had lower expression in the control condition, whereas 285 genes exhibited a discordant fold change between the two datasets. Consistent with our previous publication^15^, OTX2 silencing results in a significant decrease in genes associated with the cell cycle, MYC targets and mTORC1 signalling (Extended Data Fig. 4b). We also observed significant downregulation in genes associated with nucleocytoplasmic transport and the spliceosome (Fig. 2c).

**Fig. 3:**
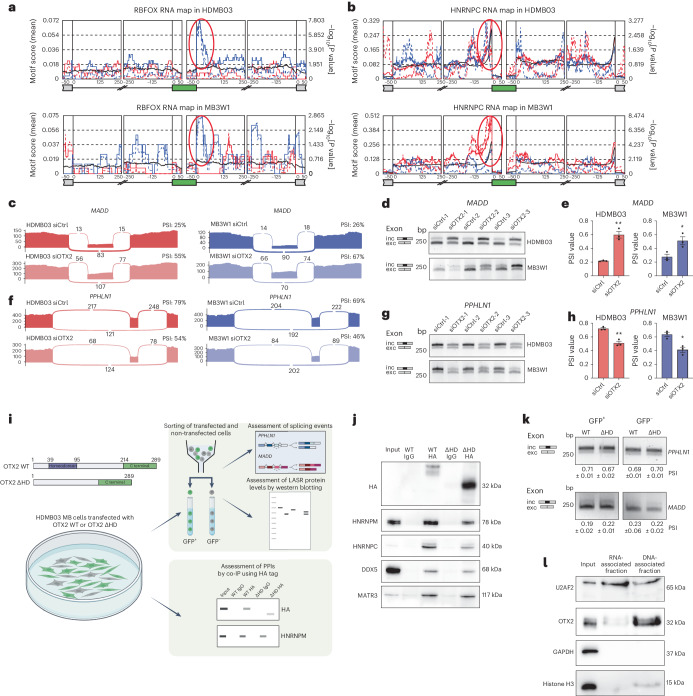
OTX2 associates with the LASR complex and regulates alternative splicing in a manner that is partially independent of the homeodomain. **a**,**b**, RBFOX (**a**) and HNRNPC (**b**) RNA maps in HDMB03 and MB3W1 tumourspheres showing significant enrichment motifs downstream or upstream, respectively, of OTX2-regulated ASEs. Wilcoxon’s rank-sum test was used to identify positions with significant differences in motif scores. Black lines represent the background set of exons. Solid red and blue lines represent up- and downregulated OTX2-regulated ASEs, respectively. Dashed red and blue lines represent −log_10_[*P* value] of motif scores versus the background. **c**, Representative Sashimi plots from RNA-seq data depicting *MADD* inclusion and exclusion reads, as well as PSI values for siCtrl and siOTX2 HDMB03 (red) and MB3W1 (blue) tumourspheres. *n* = 3 biological replicates per cell line. **d**,**e**, RT-PCR validation (**d**) and quantification (**e**) of *MADD* exon levels for siCtrl and siOTX2 tumourspheres from HDMB03 and MB3W1 cells. The error bars represent s.e.m. (*n* = 3 biological replicates per condition and cell line). Statistical significance was determined by unpaired *t*-test (**P = 0.0014 (HDMB03) and *P = 0.027 (MB3W1)). inc, included; exc, excluded. **f**, Representative Sashimi plots from RNA-seq data depicting *PPHLN1* inclusion and exclusion reads, as well as PSI values for siCtrl and siOTX2 HDMB03 (red) and MB3W1 (blue) tumourspheres. *n* = 3 biological replicates per cell line. In **c** and **f**, numbers in the Sashimi plots represent the number of junction reads. **g**,**h**, RT-PCR validation (**g**) and quantification (**h**) of *PPHLN1* exon levels for siCtrl and siOTX2 tumourspheres from HDMB03 and MB3W1 cells. The error bars represent s.e.m. (*n* = 3 biological replicates per condition and cell line). Statistical significance was evaluated by unpaired *t*-test (**P = 0.0024 (HDMB03) and *P = 0.01 (MB3W1)). **i**, Schematic depicting the sequences of the OTX2 wild type (OTX2 WT) and OTX2 lacking its DNA-binding homeodomain (OTX2 ΔHD), plus the subsequent workflow with OTX2 constructs. **j**, Co-IP showing OTX2 wild type and ΔHD interaction with select LASR proteins. *n* = 3 independent experiments with similar results. **k**, RT-PCR of *PPHLN1* (top) and *MADD* (bottom) exon levels for sorted transduced (GFP^+^) and untransduced (GFP^−^) cells. The values represent PSI values ± s.e.m. (*n* = 3 biological replicates for *PPHLN1* and *n* = 2 biological replicates for *MADD*). **l**, Subcellular fractionation demonstrating a strong presence of OTX2 in the DNA fraction and a modest presence in the RNA fraction. GAPDH and histone H3 served as controls. *n* = 3 independent experiments with similar results. HA, haemagglutinin. Panel **i** created with BioRender.com. Source data

**Extended Data Fig. 5:**
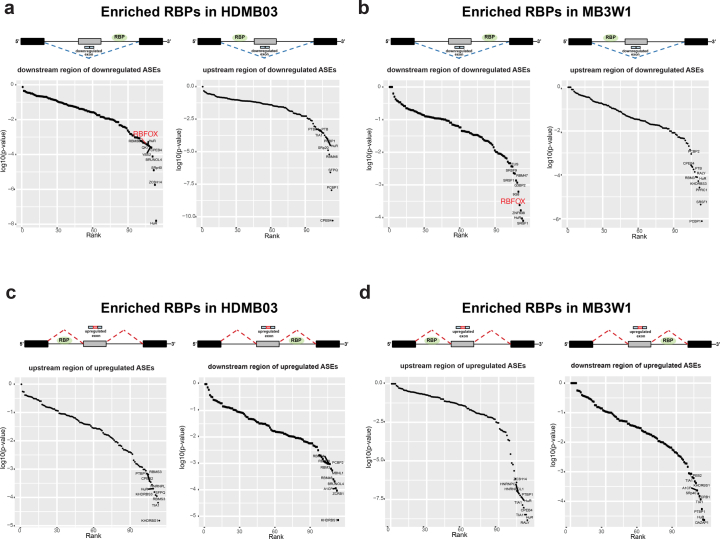
Enriched RNA binding protein motifs up- and downstream of OTX2-regulated alternatively spliced exons. **a-d**. LASR protein motifs are enriched up- and downstream of downregulated (a,b) and upregulated (c,d) alternatively spliced exons in HDMB03 (a,c) and MB3W1 (b,d). The RNA Binding Fox-1 Homolog (RBFOX) motif is highly enriched downstream of skipped OTX2-regulated alternatively spliced exons (ASEs). N = 3 biological replicated for both HDMB03 and MB3W1 samples.

A comprehensive evaluation of alternative splicing events (Fig. 2d) revealed that OTX2 silencing drives hundreds of splicing alterations in MB tumourspheres (Fig. 2e). Alternatively spliced exons (ASEs; also known as cassette exons; Supplementary Tables 5 and 6) represent the most frequent type of regulated splicing event upon OTX2 knockdown, with 40 OTX2-dependent ASEs common to both cell lines (Fig. 2f). Of these 40, 92% (37/40) exhibit directionally similar changes after OTX2 silencing (Fig. 2g,h and Extended Data Fig. 4c,d). Although the numbers of common ASEs are evenly distributed between skipped and included exons, a breakdown within each individual cell line revealed a bias towards exon inclusion (Extended Data Fig. 4e). Expanding this list to include all genes common to both cells lines harbouring ASEs, regardless of exon location, revealed a final set of 48 OTX2-regulated DSGs (Fig. 2i). These DSGs are associated with RNA processing, splicing and neurodevelopment (Fig. 2i, Extended Data Fig. 4f,g and Supplementary Table 7). Several DSGs in HDMB03 and/or MB3W1 tumourspheres have already been implicated in group 3 and/or group 4 MB tumorigenesis (that is, *SUZ12* (refs. ^15,24^), *PIEZO1* (ref. ^3^), *PVT1* (ref. ^25^) and *DST*^3^; Extended Data Fig. 4f,g and Supplementary Tables 5 and 6). Next, we performed motif analysis to evaluate enrichment of RBP motifs near OTX2-regulated ASEs using rMAPS2 software^26^ and a compilation of 110 known RBP motifs^27,28^. Interestingly, using computational methods without a priori knowledge, we found several LASR complex member motifs, including RBFOX2 and HNRNPC to be significantly enriched near OTX2-regulated ASEs (Fig. 3a,b and Extended Data Fig. 5). The RBFOX motif stood out as one of the top enriched RBP motifs downstream of ASEs downregulated by OTX2 (Fig. 3a and Extended Data Fig. 5). Notably, RBFOX2 is an important splicing regulator involved in various developmental processes^29^. Additionally, the HNRNPC motif is enriched upstream of upregulated ASEs (Fig. 3b). Our results suggest that OTX2 and LASR complex proteins may regulate alternative splicing on common targets.

**Extended Data Fig. 6:**
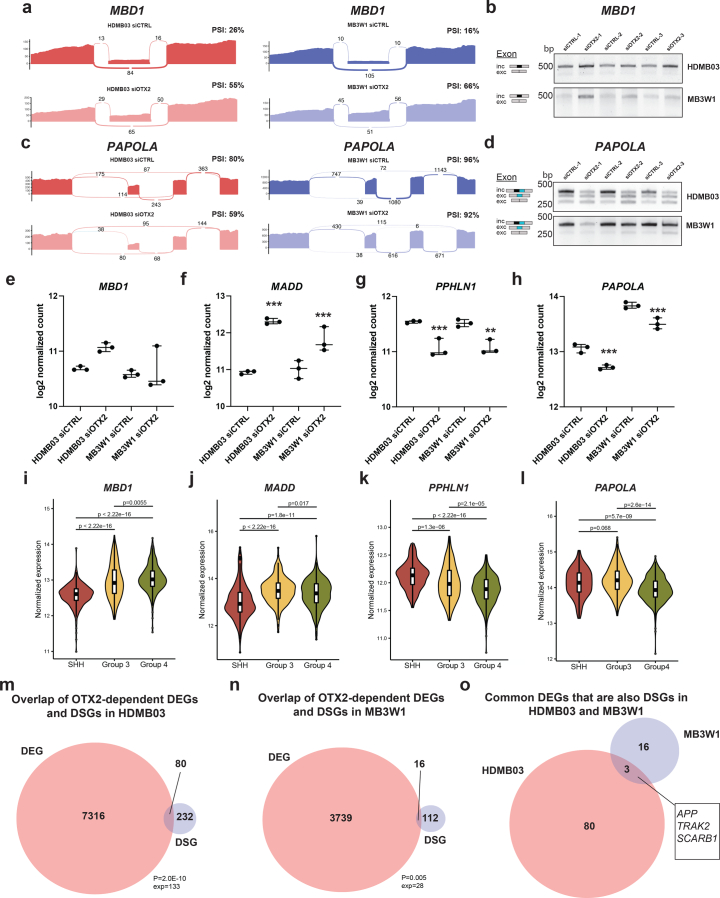
OTX2-regulated differentially spliced genes in Group 3 MB tumorspheres and across MB patient samples. **a**. Representative sashimi plots depicting *MBD1* inclusion and exclusion reads as well as percent spliced-in (PSI) values for siCTRL and siOTX2 HDMB03 (red) and MB3W1 (blue) tumorspheres. N = 3 biological replicates for each cell line. PSI (Percent Spliced In) = percentage of exon inclusion (inclusion reads/inclusion reads + exclusion reads). **b**. RT PCR validation of *MBD1* gene exon inclusion and exclusion for siCTRL and siOTX2 tumorspheres from HDMB03 and MB3W1 cells across N = 3 biological replicates. **c**. Representative sashimi plots depicting *PAPOLA* inclusion and exclusion reads as well as percent spliced-in (PSI) values for siCTRL and siOTX2 HDMB03 (red) and MB3W1 (blue) tumorspheres. N = 3 biological replicates for each cell line. PSI (Percent Spliced In) = percentage of exon inclusion (inclusion reads/inclusion reads + exclusion reads). **d**. RT PCR validation of *PAPOLA* gene exon inclusion and exclusion for siCTRL and siOTX2 tumorspheres from HDMB03 and MB3W1 cells across N = 3 biological replicates. **e-h**. Normalized counts for *MBD1* (e), *MADD* (f), *PPHLN1* (g) and *PAPOLA* (h) following OTX2 silencing in HDMB03 and MB3W1 tumorspheres obtained from RNA-seq data. While *MADD*, *PPHLN1*, and *PAPOLA* showed statistically significant expression changes following OTX2 silencing, only *MADD* levels in HDMB03 exhibited a >2-fold change and fit the criteria for significant differential expression. P < 0.01 **, P < 0.001 ***. Results were analyzed using a Wald test, with FDR correction. **i-l**. Violin plots demonstrating *MBD1* (i), *MADD* (j), *PPHLN1* (k), and *PAPOLA* (l) gene expression across MB subgroups from 763 medulloblastoma patient samples. For all plots, the center line is the median, the whiskers extend to the minima and maxima and the 25th and 75th percentiles are depicted by the bottom and top of the box, respectively. **m-o**. Overlap between differentially expressed genes (DEGs) and differentially spliced genes (DSGs) in HDMB03 (m), MB3W1 (n) and tumorspheres from both cell lines (o) following OTX2 silencing. Expected overlap for HDMB03 = 133. Expected overlap for MB3W1 = 28. Source data

**Extended Data Fig. 7:**
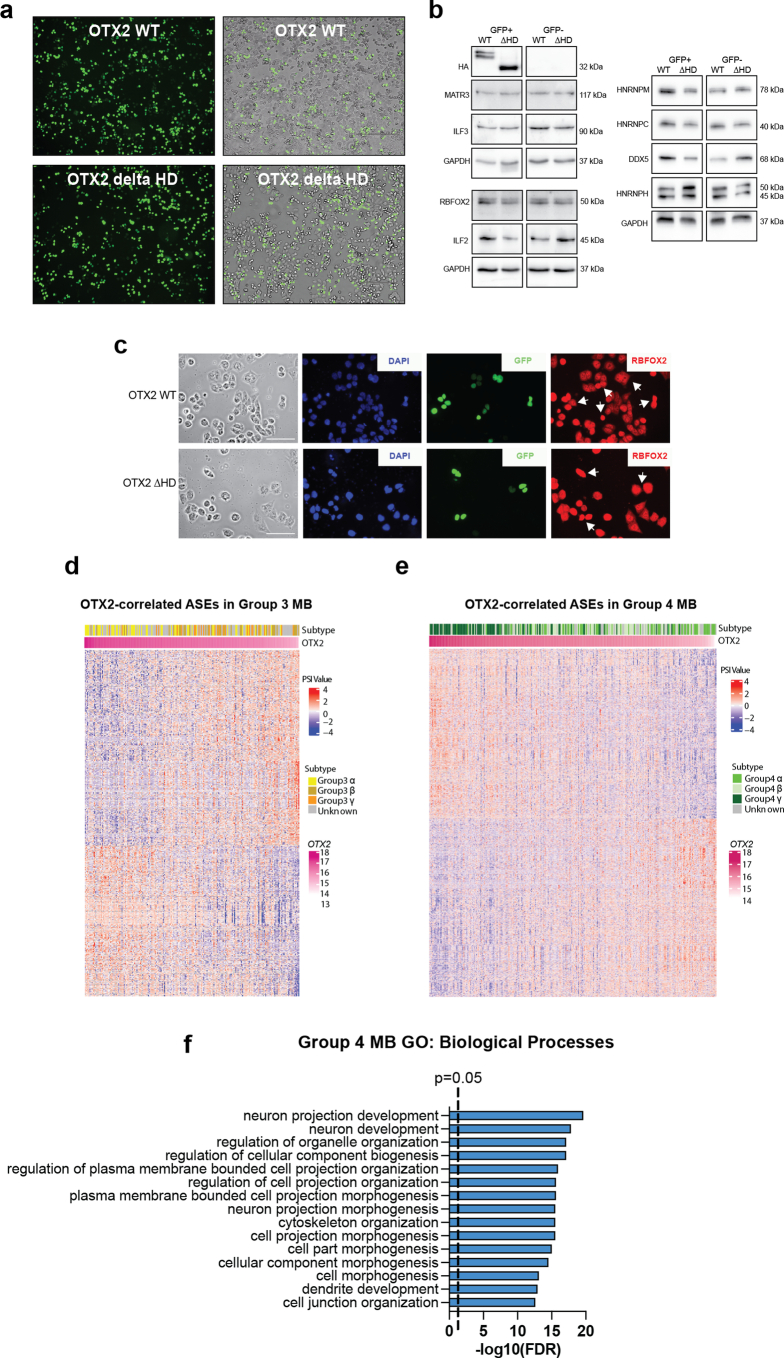
Association between deltaHD OTX2 and LASR protein levels and correlation between OTX2 and spliced genes in Group 3 MB and Group 4 MB patient samples. **a**. Representative fluorescent and brightfield images of transduction efficiency following transfection of HDMB03 MB cells with OTX2 WT or OTX2 delta HD mutant constructs. Scale bar: 300 μm. **b**. Western blots depicting LASR protein expression in sorted transduced GFP+ (WT and ΔHD) and untransduced (WT and ΔHD) HDMB03 tumorspheres. HA is used to show molecular weights of WT and truncated ΔHD OTX2 protein as well as the purity of the sort. GAPDH serves as the loading control. N = 2 independent experiments with similar results. **c**. Representative immunofluorescence images depicting RBFOX2 expression (red) in transduced (GFP+) and untransduced (GFP-) cells. DAPI is used to stain nuclei. N = 3 independent experiments with similar results. Scale bar: 75 μm. **d**. Heat map of normalized PSI values of exons significantly correlated with *OTX2* expression across 187 Group 3 MB patient samples. A Pearson correlation test was used to determine the correlation between PSI values of each individual exon and *OTX2* expression. This revealed 853 exons in 668 unique genes significantly correlated with *OTX2* (FDR < 0.01). **e**. Heat map of normalized PSI values of exons significantly correlated with *OTX2* expression across 293 Group 4 MB patient samples. A Pearson correlation test was used to determine the correlation between PSI values of each individual exon and *OTX2* expression. **f**. Enriched biological processes associated with the 1358 OTX2-correlated genes from 293 Group 4 MB patient samples. GO terms enrichment was performed with the Toppfun suite using a hypergeometric distribution and p-values adjusted using the FDR correction. Source data

We then validated splicing changes favouring either exon inclusion or exon skipping following OTX2 silencing by PCR with reverse transcription (RT-PCR; Fig. 3c–h and Extended Data Fig. 6a–d). Of the four DSGs evaluated (*MADD*, *PPHLN1*, *MBD1* and *PAPOLA*), only *MADD* exhibited a greater than twofold change in overall expression following OTX2 silencing in HDMB03 tumourspheres (Extended Data Fig. 6e–h). Expression of all four genes was often significantly higher in group 3 MB tumours relative to either SHH or group 4 primary MB tumours (Extended Data Fig. 6i–l). Comparing all DEGs and DSGs, we found minimal overlap (Extended Data Fig. 6m–o), suggesting that OTX2-mediated transcription and splicing functions may be independent for a large proportion of target genes. Although our OTX2 ChIP-seq data^15^ revealed that the majority of DSGs exhibit OTX2 peaks and binding motifs (Supplementary Table 8), only three—*APP*, *SCARB1* and *TRAK2*—were also significantly differentially expressed following OTX2 silencing (Extended Data Fig. 6o). To interrogate whether the DNA regulatory function of OTX2 is also necessary for its splicing function, we overexpressed wild-type OTX2 and OTX2 lacking its DNA-binding homeodomain in HDMB03 tumourspheres (Fig. 3i and Extended Data Fig. 7a). Consistent with the role of OTX2 as a transcription factor, we found decreased levels of some LASR proteins, such as HNRNPM and HNRNPC, in cells expressing OTX2 lacking the DNA-binding homeodomain (Extended Data Fig. 7b). Similarly, the protein levels were lower but still detected in co-IPs, and RBFOX2 localization did not change (Fig. 3j and Extended Data Fig. 7c). In support of these findings, *PPHLN1* and *MADD* splicing events are not significantly different following overexpression of OTX2 lacking its homeodomain (Fig. 3k). Next, we performed subcellular fractionation, using a previously described protocol^17,30^, to extract proteins that are associated with RNA and DNA. As expected, we found that OTX2 was strongly associated with the DNA fraction. However, the presence of OTX2 in the RNA-associated fraction indicated that OTX2 may also directly or indirectly bind RNA (Fig. 3l). Together, our results suggest that, in MB, the DNA regulatory and splicing functions of OTX2 may be partially independent.

### OTX2-regulated DSGs are expressed in the human rhombic lip

**Fig. 4:**
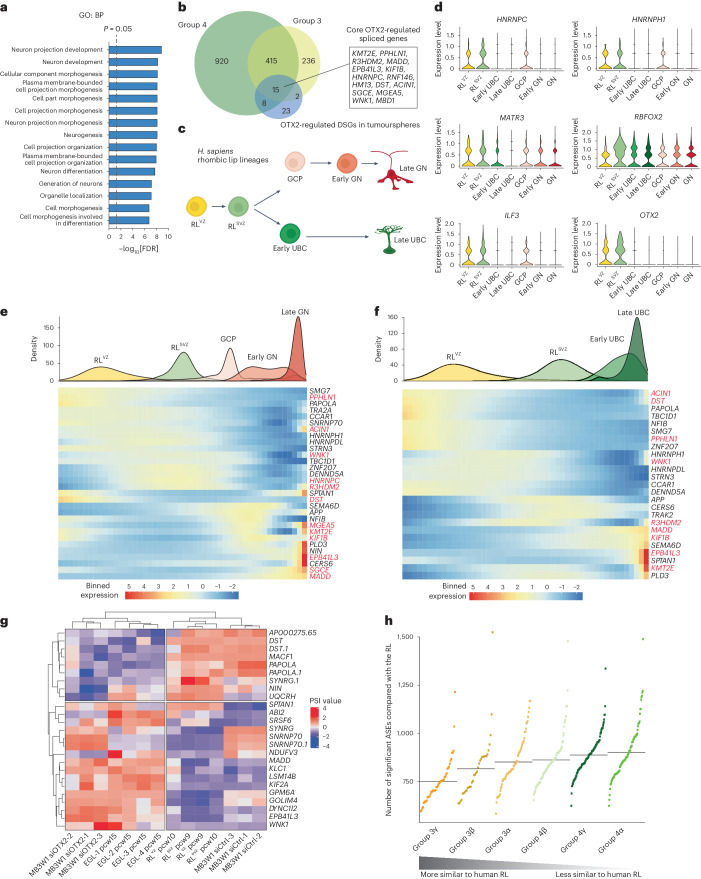
OTX2-regulated DSGs are expressed in the developing human rhombic lip. **a**, Enriched biological processes associated with the 668 OTX2-correlated DSGs from 187 samples from patients with group 3 MB. Gene Ontology term enrichment was performed with the ToppFun suite using a hypergeometric distribution and *P* values were adjusted with FDR correction. **b**, Venn diagram depicting overlap between significantly OTX2-correlated DSGs in samples from patients with group 3 and group 4 MB and the 48 significantly common OTX2-regulated DSGs in group 3 tumourspheres. **c**, Schematic of the lineages descending from the rhombic lip during human cerebellar development. In humans, but not mice or macaques, the rhombic lip splits into distinct zones termed the RL^VZ^ and RL^SVZ^. **d**, Violin plots depicting the expression levels of genes transcribing members of the LASR complex, as well as *OTX2*, in cell types of the developing human rhombic lip. **e**,**f**, Gene expression of characteristic marker genes of granule neuron (**e**) and UBC (**f**) differentiation along pseudotime following OTX2 knockdown in MB3W1 cells. In total, 30 of 48 OTX2-regulated spliced genes were significant for the granule neuron lineage, whereas 25 of 48 common OTX2-regulated spliced genes were significant for the UBC lineage. Members of the core 15 OTX2-regulated DSGs are highlighted in red. **g**, Heatmap of normalized PSI values of the significantly spliced exons in RL^VZ^/RL^SVZ^ versus EGL that were also OTX2-regulated exons in MB3W1 tumourspheres. **h**, Numbers of significant ASEs compared with the human rhombic lip (pooled RL^VZ^ and RL^SVZ^ at PCWs 9 and 10; *n* = 4) across all group 3 and group 4 MB subtypes. The dots represent individual tumours and the median per subtype is represented by a horizontal line. The MB subtypes are ordered by increasing number of significant splicing events relative to the human rhombic lip, with group 3γ tumours being the least divergent or more similar to their developmental origins. GN, granule neuron. Source data

To further explore the relevance of OTX2-regulated splicing, we expanded our findings to primary MB tumours. Using bulk RNA-seq from 187 group 3 MBs and 293 group 4 MBs (Fig. 4a,b and Extended Data Fig. 7d,e)^3^, we performed correlation analysis between the percentage spliced in (PSI) value of ASEs and *OTX2* expression. In total, we found that alternative splicing of 853 exons (in 668 genes) was highly correlated with the *OTX2* expression level in group 3 MBs (false discovery rate (FDR) < 0.01) and these results exhibited substantial overlap with group 4 MBs (430 of 668 genes; Fig. 4a,b, Extended Data Fig. 7d,e and Supplementary Table 9). Functional enrichment analyses demonstrated that these genes are strongly associated with neurodevelopmental programs in both subgroups (Fig. 4a and Extended Data Fig. 7f). Additional overlap of the 48 OTX2-regulated DSGs in our tumoursphere models revealed that 15 are also significantly correlated with *OTX2* expression in group 3 MB and group 4 MB, constituting a core set of OTX2-regulated spliced genes (Fig. 4b). The presence of our validated genes *MADD*, *MBD1* and *PPHLN1* across all datasets suggested that these DSGs may be regulators of MB tumorigenesis.

**Extended Data Fig. 8:**
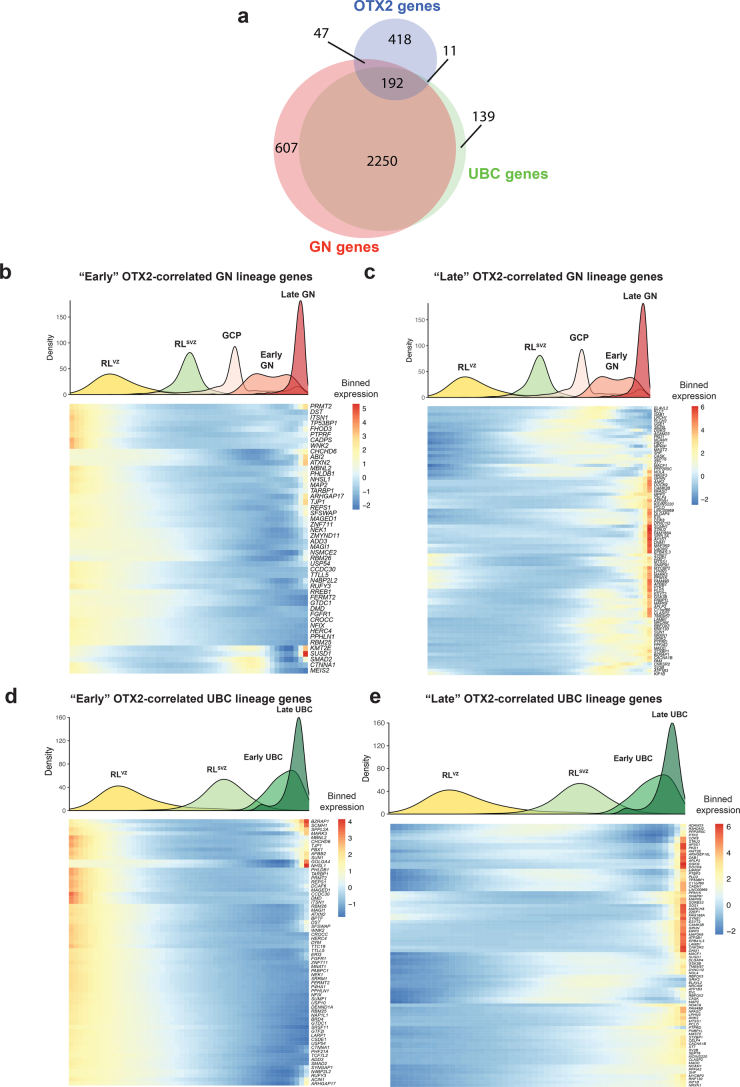
OTX2 levels are correlated with spliced genes in Group 3 MB patient samples and are associated with the developing rhombic lip. **a**. Venn diagram depicting overlap between common OTX2-correlated genes (Group 3 MB patient samples) and lineage genes from the developing rhombic lip (RL) of the cerebellum. **b-c**. Gene expression of characteristic marker genes of granule neuron (GN) differentiation along pseudotime. OTX2-correlated spliced genes in Group 3 MB tumors significant for the ‘early’ GN lineage (b) or ‘late’ GN (c) lineage. **d-e**. Gene expression of characteristic marker genes of unipolar brush cell (UBC) differentiation along pseudotime. OTX2-correlated spliced genes in Group 3 MB tumors significant for the ‘early’ UBC lineage (d) or ‘late’ UBC (e) lineage.

We recently demonstrated that MB is a cancer of the developing human rhombic lip, with OTX2 maintaining rhombic lip identity and suppressing differentiation^3^. The *Homo sapiens* rhombic lip is developmentally unique as it splits into a stem cell-enriched RL^VZ^ and progenitor-enriched RL^SVZ^, which gives rise to both unipolar brush cells (UBCs) and granule cell precursors (GCPs) residing in the external granular layer (EGL; Fig. 4c)^6,31^. Given the association between our splicing signatures and neurodevelopmental programs, we hypothesized that OTX2-regulated DSGs participate in rhombic lip specification. Interestingly, genes transcribing members of the LASR complex are highly expressed in the RL^VZ^ and RL^SVZ^ (Fig. 4d), consistent with *OTX2* expression in these regions. Of the 668 group 3 MB OTX2-correlated genes, 250 are also significantly correlated with cerebellar rhombic lip lineage pseudotime (Extended Data Fig. 8a). Approximately half are expressed in UBCs and GCPs or downstream granule neurons (designated late genes) and the other half are highly expressed in the RL^VZ^ stem cell compartment, with some extending to RL^SVZ^ progenitors (designated early genes) (Extended Data Fig. 8a–e). Moreover, pseudotime mapping of our 48 OTX2-regulated DSG tumoursphere signatures revealed a similar pattern, with 30 and 25 genes overlapping with the granule neuron and UBC lineage genes, respectively, including the majority of the 15 OTX2-regulated DSGs constituting the core set (Fig. 4e,f). Importantly, our validated DSGs represent opposite ends of the differentiation spectrum, as *PPHLN1* and *PAPOLA* are primarily expressed in the early RL^VZ^ compartment, whereas *MADD* is expressed later in the more mature lineages of UBC and granule neuron cells (Fig. 4e,f).

**Extended Data Fig. 9:**
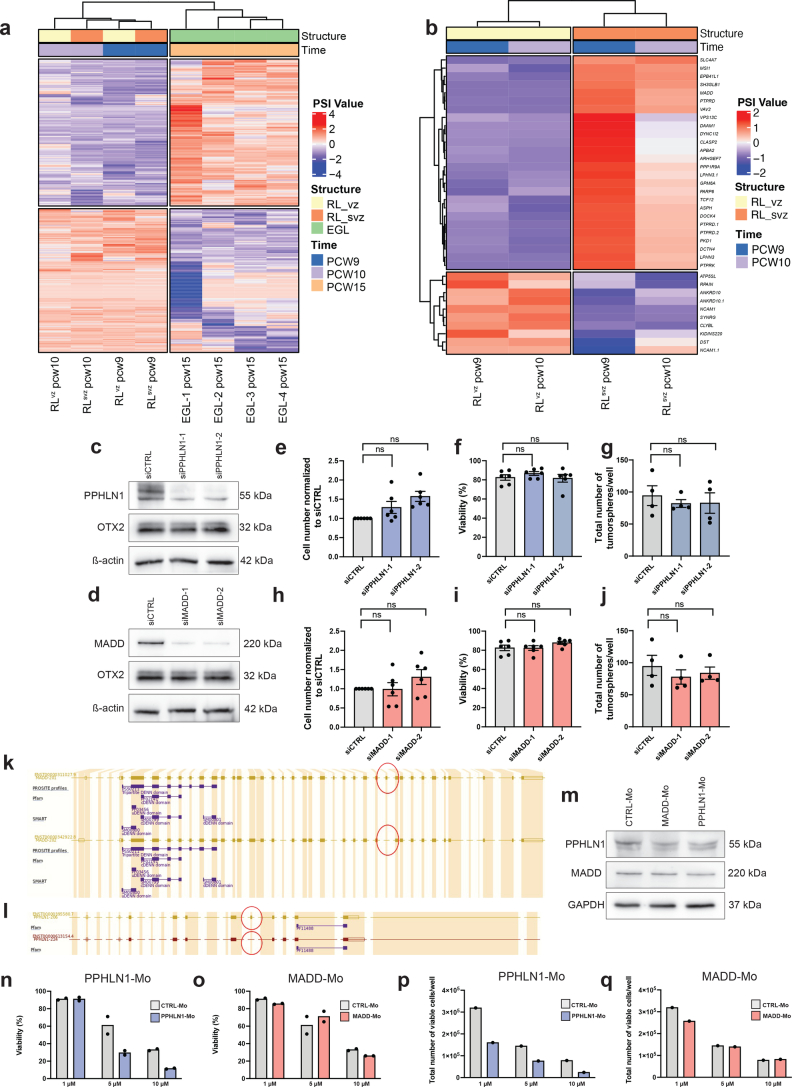
Splice blocking, but not global siRNA silencing of PPHLN1 and MADD in Group 3 MB tumorspheres impacts tumorigenic properties *in vitro.* **a**. Heat map of normalized PSI values from ASEs in RL^VZ^/RL^SVZ^ vs. the external granule layer (EGL). RL^VZ^ and RL^SVZ^ at post conception weeks (PCW) 9 and 10, N = 4 versus the EGL at PCW 15, N = 4. **b**. Heat map of normalized PSI values from ASEs in RL^VZ^ vs. the RL^SVZ^. RL^VZ^ at post conception weeks (PCW) 9 and 10, N = 2 vs. RL^SVZ^ at post conception weeks (PCW) 9 and 10, N = 2. **c-d**. Immunoblot validation of PPHLN1 (c) and MADD (d) silencing following treatment with 2 independent siRNAs or a negative control siRNA (siCTRL) for each gene. OTX2 levels are unaffected. β-actin serves as a loading control. N = 2 experiments for both PPHLN1 and MADD with similar results. **e-g**. Quantification of HDMB03 total live cell number (e), viability (f) and total number of tumorspheres (g) following 5 day treatment with siCTRL, siPPHLN1-1, or siPPHLN1-2. Error bars: SEM. Raw data were analyzed using one-way ANOVA and a Dunnett’s test for multiple comparisons. **h-j**. Quantification of HDMB03 total live cell number (h), viability (i) and total number of tumorspheres (j) following 5 day treatment with siCTRL, siMADD-1, or siMADD-2. Error bars: SEM. Raw data were analyzed using one-way ANOVA and a Dunnett’s test for multiple comparisons. **k**. MADD splicing isoforms of interest and their corresponding protein domains from PROSITE, Pfam and SMART databases. Exon of interest is highlighted in red. **l**. PPHLN1 splicing isoforms of interest and their corresponding protein domains from PROSITE, Pfam and SMART databases. Exon of interest is highlighted in red. **m**. Western blot depicting protein levels of PPHLN1 and MADD following HDMB03 tumorsphere treatment with the CTRL-Mo, MADD-Mo and PPHLN1-Mo. GAPDH serves as a loading control. **n-q**. Initial toxicity test and associated quantification of viability (n-o) (N = 2 independent experiments) and total number of viable cells (p-q) (N = 1) following HDMB03 treatment with 1, 5, or 10 μM PPHLN1-Mo (n,p) or MADD-Mo (o,q) relative to CTRL-Mo for 48 hours. Source data

As OTX2 silencing drives group 3 MB cells towards a more differentiated GCP/granule neuron phenotype^3^, we used previously published RNA-seq data^6,31^ to compare alternative splicing programs in the human rhombic lip and EGL. Of the 552 ASEs (Extended Data Fig. 9a and Supplementary Table 10) found between the rhombic lip and EGL, 25 are also OTX2-regulated ASEs in MB3W1 tumourspheres (Fig. 4g). Hierarchical clustering revealed similarities between the splicing landscapes of OTX2-silenced MB3W1 tumourspheres and EGL cells, whereas siCtrl MB3W1 tumourspheres clustered with rhombic lip cells. These results are consistent with the notion that OTX2 is a repressor of GCP/granule neuron cell fate^3^. We then explored splicing programs in the RL^VZ^ versus the RL^SVZ^ and found that *MADD* was also differentially spliced between the two subcompartments (Extended Data Fig. 9b and Supplementary Table 11), demonstrating that a lower *MADD* PSI value is associated with the stem cell-enriched RL^VZ^. Strikingly, group 3γ MBs, which are closely aligned with the stem cell-enriched primitive RL^VZ^ at the transcriptional level, are also the least divergent from the human rhombic lip at the post-transcriptional level, suggesting that this subtype shares developmental features of the human rhombic lip (Fig. 4h). Overall, these data demonstrate that group 3 MB post-transcriptional splicing programs recapitulate the developmental trajectories of the normal human hindbrain.

### Spliced variants of *PPHLN1* and *MADD* regulate cell fate decisions

**Fig. 5:**
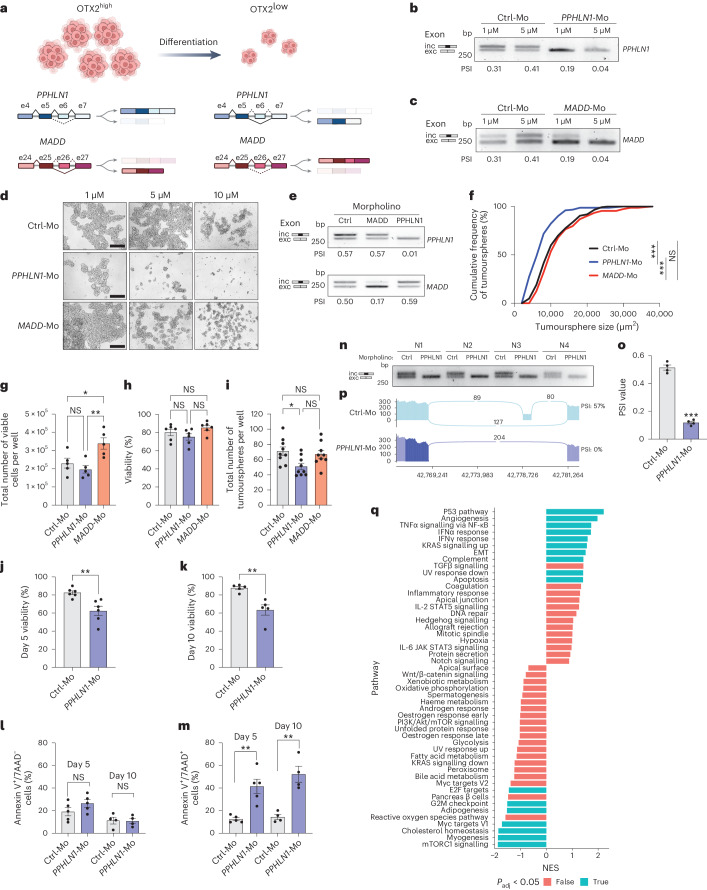
Alternative splicing of *PPHLN1* and *MADD* is associated with stem and progenitor cell states. **a**, Schematic depicting *PPHLN1* and *MADD* alternative splicing relative to OTX2 expression. **b**,**c**, RT-PCR validation of *PPHLN1* (**b**) and *MADD* (**c**) exon levels following 48 h morpholino treatment. *n* = 3 independent experiments with similar results. **d**, HDMB03 tumourspheres treated with morpholinos for 48 h. Scale bars, 300 μm. *n* = 3 independent experiments with similar results. **e**, RT-PCR validation of *PPLHN1* and *MADD* exon levels following 72 h of 1 μM morpholino treatment. *n* = 3 independent experiments with similar results. **f**, Tumoursphere size following 72 h treatment with 1 μM Ctrl-Mo (black), *PPHLN1*-Mo (blue) or *MADD*-Mo (red). Statistical significance was determined by two-sample Kolmogorov–Smirnov test (***P < 0.001). **g**–**i**, Quantification of HDMB03 live cell number (**g**), viability (**h**) and total number of tumourspheres (**i**) following 72 h (**g** and **h**) or 5 d (**i**) of treatment. The error bars represent s.e.m. (*n* = 5 (**g**), 6 (**h**) and 9 (**i**) biological replicates). Statistical significance was determined by one-way ANOVA with Tukey’s test for multiple comparison (in **g**, *P = 0.033 for Ctrl-Mo versus *MADD*-Mo and **P = 0.0070 for *MADD*-Mo versus *PPHLN1*-Mo; in **i**, *P = 0.0215 for Ctrl-Mo versus *PPHLN1*-Mo). **j**,**k**, Quantification of trypan blue staining following 5 d (**j**) or 10 d (**k**) of treatment. The error bars represent s.e.m. (*n* = 6 (**j**) and 5 (**k**) biological replicates). Statistical significance was determined by unpaired *t*-test (**P = 0.0044 (**j**) and **P = 0.0034 (**k**)). **l**,**m**, Quantification of annexin V staining (annexin V^+^/7AAD^−^ dying cells (**l**) and annexin V^+^/7AAD^+^ dead cells (**m**)) following 5 or 10 d of treatment. The error bars represent s.e.m. (for both **l** and **m**, *n* = 5 (5 d) and 4 (10 d) biological replicates). Statistical significance was determined by unpaired *t*-test (for both 5 and 10 d in **m**, **P = 0.002). **n**,**o**, Representative PCR (**n**) and quantification (**o**) of *PPHLN1* exon 6 levels following 5 d of treatment with 1 μM *PPHLN1*-Mo. The error bars represent s.e.m. (*n* = 4 biological replicates). Statistical significance was determined by unpaired *t*-test (***P < 0.001). N1–N4 represent four biological replicates. **p**, Sashimi plots of *PPHLN1* exon 6 following 1 μM *PPHLN1*-Mo treatment. Numbers in the Sashimi plots represent the number of junction reads. **q**, Gene set enrichment analysis demonstrating hallmark pathways enriched following 5 d of 1 μM *PPHLN1*-Mo versus Ctrl-Mo treatment. *n* = 4 biological replicates. IFN, interferon; EMT, epithelial-to-mesenchymal transition; IL-2, interleukin-2; NES, normalized enrichment score; NF-κB, nuclear factor-κB; NS, not significant; TGF, transforming growth factor; TNF, tumour necrosis factor; UV, ultraviolet. Source data

As pseudotime mapping identified the OTX2-regulated DSGs *PPHLN1* and *MADD* as early and late genes, respectively, we hypothesized that silencing their expression would have differential effects on group 3 MB tumourspheres. We globally knocked down *PPHLN1* and *MADD* using two independent small interfering RNAs (siRNAs) (Extended Data Fig. 9c,d). Silencing overall transcription of either gene had no effect on tumour properties (Extended Data Fig. 9e–j); thus, we turned our attention to the specific ASEs regulated by OTX2. As *PPHLN1* was predicted to be associated with stem cell state based on our tumoursphere and cerebellar datasets, we designed a custom splice-blocking morpholino to induce *PPHLN1* exon skipping (ENSG00000134283.13, exon 6), which recapitulated the post-transcriptional change observed following OTX2 silencing (Figs. 3f–h and 5a,b). A splice-blocking morpholino was also designed to induce *MADD* exon skipping (ENSG00000110514.14, exon 26); however, OTX2 silencing favours MADD exon retention (Figs. 3c–e and 5c); thus, we predicted that this morpholino would either increase or have no effect on group 3 MB tumourspheres. Importantly, the total number of bases per skipped exon (57 for ENSG00000134283.13, exon 6 (*PPHLN1*) and 63 for ENSG00000110514.14, exon 26 (*MADD*)) was divisible by 3; therefore, the morpholinos were not expected to change the open reading frame. In addition, *PPHLN1* exon 6 and *MADD* exon 26 were not predicted to encode a functional domain (Extended Data Fig. 9k,l).

**Fig. 6:**
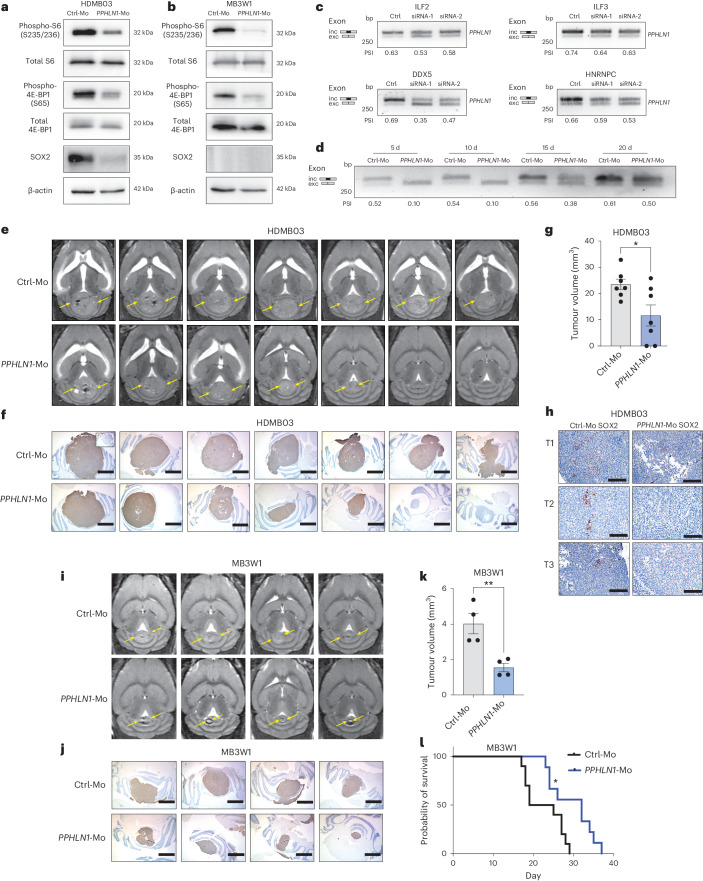
Skipping of *PPHLN1* exon 6 inhibits stem cell properties and impairs tumour growth in vivo. **a**,**b**, Immunoblots of HDMB03 (**a**) and MB3W1 (**b**) tumourspheres following 5 d of 1 μM *PPHLN1*-Mo treatment. β-actin, total S6 and total 4E-BP1 served as loading controls. *n* = 2 independent experiments per cell line with similar results. **c**, RT-PCR validation of *PPHLN1* exon 6 levels following 72 h treatment with siCtrl or siRNAs for LASR complex members in HDMB03 tumourspheres. The experiment was repeated three times with similar results. **d**, Exon skipping, evaluated at days 5 (passage 1), 10 (passage 2), 15 (passage 3) and 20 (passage 4), of HDMB03 tumourspheres treated with 1 μM *PPHLN1*-Mo on day 0. The experiment was repeated twice with similar results. **e**, MRI images of representative tumours derived from Ctrl-Mo- and *PPHLN1*-Mo-treated HDMB03 cells following a 5 d pre-treatment. The yellow arrows denote tumour margins. **f**, Representative immunohistochemical staining for anti-mitochondrial antibodies in formalin-fixed, paraffin-embedded tissue sections derived from NOD-SCID mice injected with Ctrl-Mo- and *PPHLN1*-Mo-treated HDMB03 MB cells. *n* = 7 mice per treatment group. Scale bars, 1,550 μm. Inset represents secondary only negative control. **g**, Tumour volume in mice transplanted with Ctrl-Mo- and *PPHLN1*-Mo-treated HDMB03 cells. The error bars represent s.e.m. (*n* = 7). Statistical significance was determined by unpaired *t*-test (*P = 0.0206). **h**, Representative SOX2 immunohistochemical staining in sections derived from independent NOD-SCID mice injected with Ctrl-Mo- (left) or *PPHLN1*-Mo-treated (right) HDMB03 MB cells. Scale bars, 150 μm. *n* = 3 per group. **i**, MRI images of representative tumours derived from Ctrl-Mo- and *PPHLN1*-Mo-treated MB3W1 cells. The yellow arrows denote tumour margins. **j**, Representative immunohistochemical staining for anti-mitochondrial antibodies in sections derived from NOD-SCID mice injected with Ctrl-Mo- and *PPHLN1*-Mo-treated MB3W1 MB cells. *n* = 4 mice per treatment group. Scale bar, 1,550 μm. **k**, Tumour volume in NOD-SCID mice transplanted with Ctrl-Mo- and *PPHLN1*-Mo-treated MB3W1 MB cells. The error bars represent s.e.m. (*n* = 4). Statistical significance was determined by unpaired *t*-test (**P = 0.0069). **l**, Kaplan–Meier curves following transplantation of NOD-SCID mice with MB3W1 cells (*n* = 10 for Ctrl-Mo and *n* = 9 for *PPHLN1*-Mo). Statistical significance was determined by log-rank test (*P = 0.0152). Source data

**Extended Data Fig. 10:**
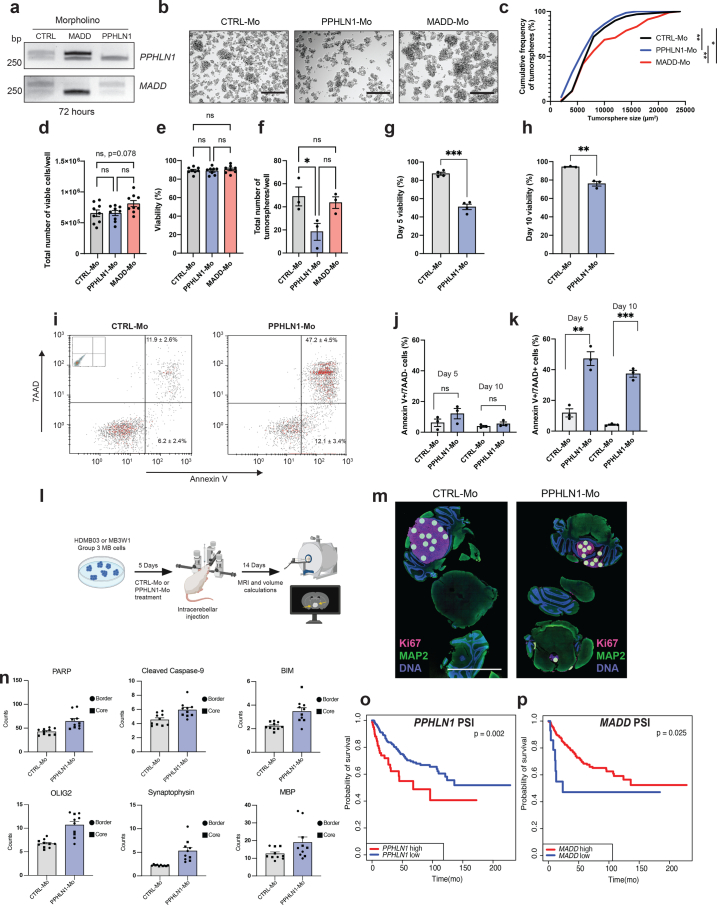
A PPHLN1-Mo and MADD-Mo have different effects on MB3W1 Group 3 MB tumor properties. **a**. RT PCR validation of specific *PPLHN1* or *MADD* gene exon exclusion following 72 hour 5 μM PPHLN1-Mo or MADD-Mo treatment respectively relative to CTRL-Mo in MB3W1. N = 3 experiments with similar results. **b**. Representative images of MB3W1 tumorspheres treated with 1 μM control (CTRL-Mo) (left), PPHLN1 (PPHLN1-Mo) (middle) or MADD (MADD-Mo) (right) morpholinos for 5 days. Scale bar: 300 μm. **c**. Cumulative frequency distribution of MB3W1 tumorsphere size following 5 day treatment with 1 μM CTRL-Mo (black), PPHLN1-Mo (blue) or MADD-Mo (red). Tumorsphere size was analyzed using 2-sample Kolmogorov–Smirnov tests. p < 0.05*, p < 0.01**. CTRL-Mo vs. PPHLN1-Mo, p = 0.0044; CTRL-Mo vs. MADD-Mo, p = 0.0442; PPHLN1-Mo vs. MADD-Mo, p = 0.0071. **d–f**. Quantification of MB3W1 total live cell number (d), viability (e) and total number of tumorspheres (f) following 72 hour (d-e) or 5 day (f) treatment with CTRL-Mo, PPHLN1-Mo or MADD-Mo. Error bars: SEM. p < 0.05*, p = 0.0475. Results were analyzed using one-way ANOVA and a Tukey’s test for multiple comparisons. **g–h**. Quantification of MB3W1 tumorsphere viability by Trypan Blue staining following 5 day (g) or 10 day (h) treatment with PPHLN1-Mo relative to CTRL-Mo. Error bars: SEM. p < 0.001***. For G, p < 0.0001. For H, p = 0.0019. Results were analyzed using unpaired t-tests. **i**. Representative flow cytometry plots depicting Annexin V/7AAD staining following CTRL-Mo (left) and PPHLN1-Mo (right) treatment of MB3W1 tumorspheres. Inset: unstained control. **j-k**. Quantification of MB3W1 tumorsphere viability by Annexin V staining. j: Annexin V+/7AAD- dying cells, k: Annexin V+/7AAD+ dead cells following 5 day or 10 day treatment with PPHLN1-Mo relative to CTRL-Mo. Error bars: SEM. For 5 days, p = 0.0025, and for 10 days, p = 0.0001. Results were analyzed using unpaired t-tests. **l**. Schematic depicting PPHLN1-Mo treatment of HDMB03 or MB3W1 tumorpsheres followed by intracerebellar injection into NOD SCID mice and subsequent analyses of tumor volume. **m**. Immunofluorescent images depicting regions of interest (ROIs) from the CTRL-Mo and PPHLN1-Mo samples utilized for multiplex protein analyses. Samples were stained for MAP2 (green), Ki67 (pink) and Syto13 (blue) for tumor visualization. Scale bar: 5 mm. **n**. Barplots depicting levels of select cell death and differentiation proteins across ROIs from the CTRL-Mo and PPHLN1-Mo sample. Error bars: SEM. n = 10 selected ROIs from 1 representative slide for each treatment arm. **o-p**. Kaplan-Meier curve of *PPHLN1* exon 6 (chr12:42778741-42778798) (o) and *MADD* exon 26 (chr11:47330530-47330593) (p) in Group 3 and Group 4 MB tumors showing significant differences in overall survival. Tumors were separated by k-means clustering based on their PSI value. Panel **l** created with BioRender.com. Source data

The *PPHLN1* (*PPHLN1*-Mo) and *MADD* morpholinos (*MADD*-Mo) were highly effective at inducing exon skipping (Fig. 5b,c) without affecting protein levels (Extended Data Fig. 9m). In keeping with our hypothesis, *PPHLN1*-Mo, but not *MADD*-Mo induced rapid changes in HDMB03 cell numbers and cell death in a dose-dependent manner (Extended Data Fig. 9n–q). Morpholino concentrations at 5 µM and higher were toxic (Fig. 5d); thus, we performed more extensive characterization at 1 µM (Fig. 5e–m). *PPHLN1*-Mo resulted in smaller sizes and reduced numbers of HDMB03 tumourspheres (Fig. 5f–i). Significant decreases in cell viability were also observed following extended 5- and 10-d treatments (Fig. 5j–m). In contrast, *MADD*-Mo increased cell numbers but had no significant effect on other tumour properties (Fig. 5f–i). Similar results were obtained following treatment of MB3W1 tumourspheres (Extended Data Fig. 10a–k), as *PPHLN1*-Mo also significantly inhibits tumour properties, but *MADD*-Mo induces only a modest but significant increase in tumoursphere size (Extended Data Fig. 10c). To examine the molecular effects of inhibitory *PPHLN1*-Mo treatment on group 3 MB cells, we carried out RNA-seq on HDMB03 cells treated with 1 µM *PPHLN1*-Mo for 5 d in culture (Fig. 5n–p). Skipping of *PPHLN1* exon 6 after *PPHLN1*-Mo treatment was confirmed by RT-PCR and Sashimi plot (Fig. 5n–p). *PPHLN1*-Mo treatment resulted in significant upregulation of genes associated with hallmark apoptotic and TP53 pathways (Fig. 5q and Supplementary Table 12) and significant downregulation of genes associated with mTORC1 signalling and E2F targets, consistent with the results observed in OTX2-silenced cells (Fig. 5q)^15^. We validated a reduction in mTORC1 activity (as measured by phospho-4E-BP1 and phospho-S6 levels), as well as the stem cell marker SOX2, by immunoblot (Fig. 6a,b).

Finally, as OTX2 binds LASR proteins and we predicted that they regulate common splicing programs, we expected that knocking down LASR-related genes individually would result in similar *PPHLN1* splicing changes. Indeed, silencing of LASR complex members altered *PPHLN1* splicing in a manner similar to that observed following OTX2 knockdown (Fig. 6c). Our data underscore the functional relevance of OTX2-regulated DSGs, such as *PPHLN1* and *MADD*, in regulating group 3 MB cell fate.

### *PPHLN1*-Mo inhibits tumour growth in vivo

Given the reduction in group 3 MB tumoursphere size and number following one-time *PPHLN1*-Mo treatment and the sustained exon skipping over 15 d in vitro (Fig. 6d), we hypothesized that *PPHLN1*-Mo would be sufficient to impair tumour initiation and growth in vivo. HDMB03 tumourspheres were treated with 1 μM *PPHLN1*-Mo for 5 d before dissociation and injection into the cerebellum of NOD-SCID mice (Extended Data Fig. 10l). Remarkably, magnetic resonance imaging (MRI) and volume analyses 14 d after transplantation revealed a significant reduction in the growth of *PPHLN1*-Mo-treated cells (Fig. 6e–g), with concomitant depletion of SOX2^+^ cell clusters (Fig. 6h), suggesting a role in inhibiting stem cell properties. Similarly, MB3W1 cells pre-treated with *PPHLN1*-Mo also exhibited a significantly reduced tumour burden (Fig. 6i–k), a significant increase in survival (Fig. 6l) and elevated cell death and differentiation proteins in a representative individual sample from each group (Extended Data Fig. 10m,n and Supplementary Table 13).

**Fig. 7:**
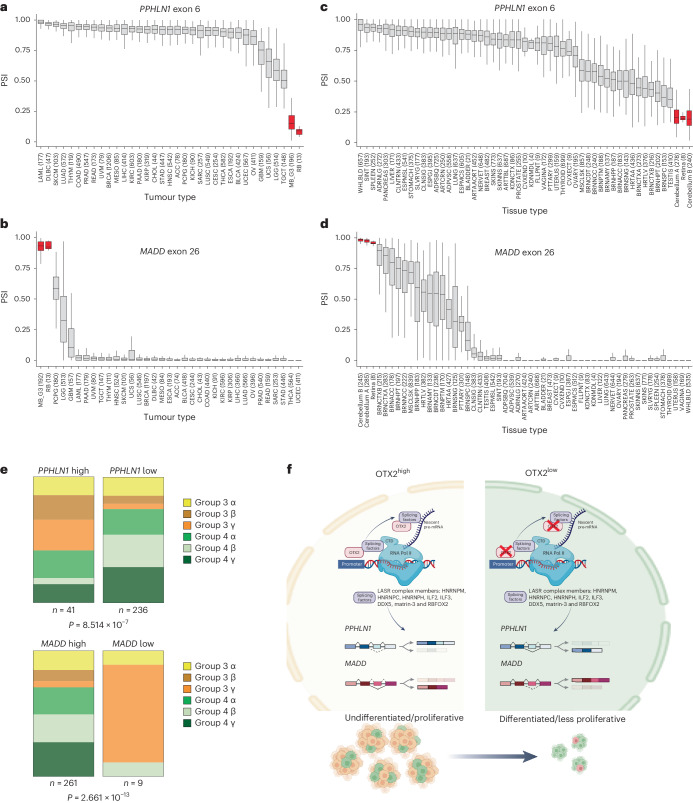
Group 3 MB and retinoblastoma *PPHLN1* and *MADD* alternative splicing patterns are similar. **a**–**d**, PSI distributions of *PPHLN1* exon 6 (**a** and **c**) and *MADD* exon 26 (**b** and **d**) across TCGA tumour types (**a** and **b**) and GTEx tissues (**c** and **d**). The results for group 3 MB (MB_G3), retinoblastoma (RB), cerebellum and retina are highlighted in red. The numbers represent the number of samples per tumour (**a** and **b**) or tissue type (**c** and **d**) in which the exon of interest was detected. Box plots show the median with IQR and whiskers represent 1.5-fold IQR. The points outside this range were outliers and are not represented. A full list of defined abbreviations is provided in Supplementary Table 14. **e**, Breakdown of *PPHLN1* exon 6 and *MADD* exon 26 PSI values using *k*-means clustering (*k* = 2) in group 3 and group 4 MB subtypes. Statistical significance was determined by chi-squared test. **f**, Proposed model of OTX2-mediated regulation of group 3 MB alternative splicing. OTX2 affects this process indirectly (through traditional DNA binding to alter the expression of splicing factors) and directly (through protein interactions with the LASR complex). This leads to alternative splicing of genes associated with cerebellar development, such as *PPHLN1* and *MADD*, in favour of isoforms that perpetuate the undifferentiated stem cell state. CTD, carboxy-terminal domain. Panel **f** created with BioRender.com.

Next, we examined the *PPHLN1* (exon 6, chr12:42778741–42778798) and *MADD* (exon 26, chr11:47330530–47330593) splicing patterns across a variety of tumour types (The Cancer Genome Atlas (TCGA)) and normal tissues (Genotype-Tissue Expression (GTEx)) (Fig. 7a–d). Of note, there are no group 3 MB^3^ or retinoblastoma^32^ samples in the TCGA database and no retina^33^ samples in the GTEx database. We therefore analysed them independently to compare PSI values. Group 3 MB and retinoblastoma (Fig. 7a,b and Supplementary Table 14), along with the normal cerebellum and retina (Fig. 7c,d and Supplementary Table 14)—both of which are known to express high levels of OTX2—exhibited unique *PPHLN1* and *MADD* splicing patterns. The relationship to the retina is intriguing, as recent studies have shown that group 3 transcriptomes display an aberrant photoreceptor program that may be derived from their rhombic lip origin^4^. Further breakdown of group 3 and group 4 MBs by *PPHLN1* and *MADD* PSI scores revealed that the *PPHLN1* high/*MADD* low PSI signature observed in our OTX2 siCtrl tumourspheres was associated with the more aggressive and primitive group 3γ subtype (Fig. 7e and Supplementary Table 15). Consistent with these findings, we generated Kaplan–Meier curves by stratifying patients with group 3 and group 4 MB into two groups using *k*-means clustering: tumours with high and low inclusion of either *PPHLN1* exon 6 or *MADD* exon 26. Patients with high inclusion levels of *PPHLN1* exon 6 and low inclusion levels of *MADD* exon 26 exhibited significantly worse survival times (Extended Data Fig. 10o,p). Therefore, following OTX2 knockdown, a splicing shift in favour of *PPHLN1* low/*MADD* high PSI scores is consistent with differentiation from a stem cell towards a progenitor cell state. OTX2-regulated DSGs help to retain the embryonic transcriptome and perpetuate the undifferentiated state of group 3 MBs.

## Discussion

An increasing number of studies have identified critical regulatory roles for DNA-binding proteins beyond transcription^34^. Although it has been known for over two decades that transcription factors can affect splicing indirectly by influencing RNA PolII elongation rates^35^, more recent studies have proposed that transcription factors can modulate splicing directly through pre-mRNA imprinting. This concept describes how transcription factors can co-transcriptionally associate with nascent transcripts, thereby affecting all downstream processes including pre-mRNA splicing, mRNA export and translation^34,36,37^. Our data highlight an unexpected role for OTX2 in mediating alternative splicing in group 3 MB (Fig. 7f). OTX2 affects this process through protein interactions with the LASR complex to regulate genes associated with cell fate decisions. These data are reinforced by the enrichment of LASR binding motifs around OTX2-regulated spliced exons, the oncogenic role of LASR members in our models, the stabilization of RBFOX2 nuclear localization by OTX2 and the presence of OTX2 in the RNA-associated fraction. The mechanisms are partially independent of the OTX2 DNA-binding domain in our group 3 MB model. We have considered the possibility that high endogenous levels of OTX2 in group 3 MB cells may be contributing to the effects. However, as OTX2 is known to dimerize^38^ and this requires the homeodomain^38^, interference from endogenous OTX2 in our ΔHD co-IP samples was probably minimal. OTX2–LASR protein interactions may need to be completely abolished to observe downstream splicing effects. In addition, future studies will test the effect of the full length wild-type OTX2 construct on splicing events in cells that do not express such high endogenous OTX2 levels. Collectively, our results suggest that OTX2 and LASR complex proteins may coordinate to regulate alternative splicing of common targets that sustain a stem cell splicing program. This is important, as the LASR complex is not a fixed splicing entity, but exhibits modular binding configurations that substantially increase the number of target genes and subsequent effects on cell function^18^. We propose that OTX2 serves as a splicing co-factor or adaptor that may influence recruitment of the LASR complex and other splicing regulators.

In addition to transcription factors, histone modifications can also regulate splicing through recruitment of splicing factors and interactions with chromatin adaptor proteins^39–43^. Interestingly, the LASR complex member RBFOX2 can interact with proteins of Polycomb repressor complex 2 (ref. ^44^), which catalyses trimethylation of histone H3 on lysine 27 (H3K27me3)^45^. Furthermore, our TurboID identified the histone–lysine *N*-methyltransferase SETDB1 as a direct OTX2 interacting partner (Fig. 1b). SETDB1 is recruited to the multiprotein human silencing hub complex, which includes PPHLN1 (refs. ^46,47^). As we and others have demonstrated that OTX2 modulates the chromatin landscape^12,15,24^ and can directly interact with Polycomb repressor complex 2 members^15^, future work will examine the interplay between OTX2, histone modifications such as H3K27me3, the human silencing hub complex and alternative splicing in controlling group 3 MB cell self-renewal and differentiation.

Previous studies examining the OTX2 interactome have identified putative splicing/RNA processing partners in both the retina^48^ and choroid plexus^49^. We have uncovered a gene regulatory layer in group 3 MB in which spliced variants of OTX2-regulated rhombic lip lineage genes significantly alter cell fate decisions. The connections between OTX2-regulated exons and early cerebellar developmental states are consistent with our recently published findings demonstrating a critical role for OTX2 in retaining rhombic lip identity^3^. The identification of functionally relevant, OTX2-regulated spliced variants of early (*PPHLN1*) and late (*MADD*) rhombic lip lineage genes adds complexity to this model, as we now demonstrate a definitive role for alternative splicing program alterations in MB tumour progression. Indeed, our *PPHLN1* high/*MADD* low PSI signature is associated with the most aggressive and primitive group 3γ subtype. Given the rapid tumour progression of our representative *MYC*-amplified group 3 MB models, the decreased tumour growth and increased survival following one-time treatment with *PPHLN1*-Mo is encouraging. Furthermore, RNA-seq of *PPHLN1*-Mo-treated cells identified significant downregulation of genes associated with mTORC1 signalling. We previously demonstrated that OTX2 regulates mTORC1 signalling in group 3 MB, and treatment with mTORC1 inhibitors significantly extended survival in xenograft models^15^. Combinatorial therapeutic approaches utilizing *PPHLN1*-Mo and mTORC1 inhibitors may lead to further decreases in group 3 MB tumour growth.

Although splicing defects are commonly observed in adult cancers, the numbers of studies focusing on alternative splicing in MB are limited^50–52^ and the functional role of alternative splicing in the most aggressive MBs has not been explored. Through its traditional role as a transcription factor, OTX2 drives the expression of some LASR-related genes, then subsequently binds these splicing factors, either directly or indirectly, to mediate alternative splicing of genes that modulate group 3 MB progression and are associated with rhombic lip development. Our study demonstrates that OTX2-mediated alternative splicing is a key determinant of cell fate decisions and highlights alternative splicing events as potential therapeutic targets to drive group 3 MB differentiation.

## Methods

### Animal ethics

All of the animal studies were approved by the Animal Care Committee at the University of Manitoba (protocol AUP-22-005).

### Cell culture

The HDMB03 and MB3W1 group 3 MB cell lines were kindly provided by Milde et al.^53^ and Wölfl and colleagues^54^, respectively. Short tandem repeat profiling (American Type Culture Collection) was used to authenticate cell lines in 2021. For tumoursphere formation, both cell lines were dissociated into single-cell suspensions. HDMB03 cells were then resuspended in StemPro NSC Serum-Free Medium (Life Technologies) and MB3W1 cells were resuspended in stem cell media (DMEM/F-12, 2% B27, 1% MEM Vitamin Solution, 20 ng ml^−1^ basic fibroblast growth factor, 20 ng ml^−1^ epithelial growth factor, 40 U ml^−1^ penicillin and 40 μg ml^−1^ streptomycin). Suspensions were plated at clonal density (for HDMB03, three cells per μl; for MB3W1, five cells per μl) in either 6- or 24-well ultra-low-attachment plates. Tumourspheres were incubated undisturbed at 37 °C under 5% CO_2_ for either 3 or 5 d, counted and then measured. Tumoursphere number (5 d), tumoursphere size (3 or 5 d), cell viability (3 d) and total cell number (5 d) were evaluated. ImageJ (Fiji) was used to evaluate individual tumoursphere size. The results were displayed as the cumulative frequency distribution of the tumoursphere area. Only tumourspheres greater than 25 μm in diameter were included in these analyses^55^. For morpholino treatment, a custom splice-blocking morpholino to induce exon skipping was designed for *PPHLN1* (ENSG00000134283.13, exon 6; 5′-AGAGTCTAGCTCAAAACTCACCTCT-3′) and *MADD* (ENSG00000110514.14, exon 26; 5′-GTCCCTTCCTGCCAATTTGAGAGCA-3′). Concentrations of 1, 5 or 10 μM in double-distilled water were added to single-cell suspensions and then cultured in ultra-low-attachment plates. An Annexin V Apoptosis Detection Kit (Annexin V-PE; BD Biosciences) was used to evaluate cell death, as previously described^15,56^.

### OTX2 siRNA silencing and RNA processing

Silencer Select siRNAs 9931 and 9932 (Life Technologies) were used at 30 nM to knockdown OTX2 expression in group 3 cells, as previously described^13,15^. A scramble (non-silencing) sequence was used as a negative control. For bulk RNA-seq, OTX2 was silenced in tumourspheres from three independent biological replicates of HDMB03 and MB3W1 cells and silencing was confirmed by western blot 3 d following transfection, as described^13,15^. Total RNA was previously extracted using a Norgen RNA extraction kit (Norgen Biotek) followed by bulk RNA-seq (paired end; 150 base pairs) targeting 100 million total reads for adequate depth of coverage for splicing analyses. Sequencing was performed at the Ottawa Hospital Research Institute/StemCore laboratories as described^3^. The raw data generated were previously deposited in the Gene Expression Omnibus (GEO) database under the access code GSE189238.

### RNA-seq, processing and analysis

Raw reads were mapped to the human genome using the hg19 reference with the STAR aligner (version 2.5.2b)^57^. Picard tools and SAMtools were used to remove PCR-duplicated reads and low-mapping-quality reads (mapping quality < 20), respectively. rMATS (version 3.0.9)^58^—an event-based tool—was used to identify differentially spliced events. We analysed five distinct alternative splicing events using rMATS: mutually exclusive exons, retained introns, skipped exons, alternative 3′ splice sites and alternative 5′ splice sites. Briefly, rMATS features a count-based model that calculates PSI scores among replicates using spliced reads and reads that map to the exon body. To call statistically significant splicing events, each splicing event had to: (1) have a ΔPSI of >10%; (2) be supported by at least 15 unique reads; and (3) have an FDR of <0.05. For gene expression analysis, aligned reads were counted using htseq-count version 0.6.1p1 (ref. ^59^) and normalized according to the DESeq size factors method^60^. To call DEGs, we used a fold change of ≥2 and an FDR of <0.05. Statistical analysis was performed and plots were generated using R environment version 3.6.0. Sashimi plots were generated using ggsashimi.py script^61^ with the parameter --min-coverage 5. We performed motif analyses to evaluate the enrichment of RBP motifs enriched near OTX2-regulated ASEs using a compilation of 110 known RBP motifs from the literature^27,28^. Briefly, we used rMAPS^62^ to identify known RBP motifs that were significantly enriched in OTX2-regulated ASEs compared with control exons (background). rMAPS examines adjacent intronic and exonic sequences and counts the number of times the motif matches each sequence to compute an enrichment score.

To explore the splicing landscape of group 3 and group 4 MB, we examined previously published bulk RNA-seq results for group 3 and group 4 tumours^3^. Briefly, we excluded exons that were not expressed in at least 25% of samples and excluded 5% of the total number of samples that had the least number of detected exons. We performed correlation-based analysis between OTX2 expression and PSI values and applied FDR correction. We considered statistically significant exons that had an adjusted *P* value of <0.01.

To determine whether OTX2-regulated exons recapitulate the developmental origins of group 3 and group 4 tumours, we used previously published cerebellar bulk RNA-seq data^6,31^ for the rhombic lip (RL^VZ^ and RL^SVZ^ at post-conception weeks (PCWs) 9 and 10; *n* = 4) and EGL (at PCW 15; *n* = 4). We also compared the splicing profile of each group 3 and 4 MB tumour with that of the human rhombic lip (pooled RL^VZ^ and RL^SVZ^ at PCWs 9 and 10; *n* = 4). We applied the same filters as described above. Because the depth of coverage for each tumour can impact the number of significant ASEs, only exons that were expressed in all tumours were retained (an event needs to be supported by at least 15 unique reads) for subsequent analyses. Briefly, the list of exons expressed in each tumour was extracted. Then, we removed samples that had too few or too many expressed exons and considered them as outliers (samples that were outside box plot whiskers: *n* < Q1 – 1.5 × interquartile range (IQR) or *n* > Q3 + 1.5 × IQR). Next, we compared the lists of expressed exons for each tumour individually to determine the minimal number of exons expressed across all tumours (*n* = 15,342). This list of minimal expressed exons was then compared with the list of significant ASEs for each group 3 and group 4 tumour, resulting in the number of events that were significantly spliced relative to the human rhombic lip. For the TCGA and GTEx datasets, PSI values were extracted from the TCGA SpliceSeq database^63^ and SnapMine^64^, respectively. Bulk RNA-seq data from retinoblastoma and normal retina samples were accessed from GEO under the accession codes GSE196420 (ref. ^32^) and GSE99248 (ref. ^33^), respectively.

For RNA-seq on *PPHN1*-Mo-treated cells, HDMB03 cells were treated with 1 μM Ctrl-Mo or *PPHLN1*-Mo for 5 d. Cells were collected and RNA was extracted using a Total RNA purification kit (Norgen Biotek). RNA-seq was performed as described above using the same alignment parameters and analysis tools.

We performed functional and pathway enrichment analysis on genes exhibiting significant changes in expression following OTX2 silencing; this was done separately for genes up- and downregulated following knockdown. Enrichment analysis was performed using the ClusterProfiler tool^65^ to look at the enrichment of Gene Ontology molecular function and biological process annotations and Kyoto Encyclopedia of Genes and Genomes (KEGG) pathways. Within each group of annotations, enrichment was calculated by comparison with the hypergeometric distribution (equivalent to a one-tailed Fisher’s exact test). Multiple testing correction was performed using the Benjamini–Hochberg approach. The KEGG pathway data were obtained from the KEGG website on 11 February 2022.

### RT-PCR validations of select DSGs

The DSGs *PPHLN1*, *PAPOLA*, *MADD* and *MBD1* were selected for further validation by RT-PCR. Total RNA was extracted from both HDMB03 and MB3W1 tumourspheres using a Total RNA Purification Kit (Norgen Biotek). First-strand complementary DNA was generated using the SuperScript III First-Strand Synthesis kit (Life Technologies). PCR was performed using the MiniAmp Plus Thermal Cycler (Applied Biosystems, Thermo Fisher Scientific) with the following parameters: 98 °C for 10 s, 55 °C for 5 s and 72 °C for 5 s over 30–35 cycles. The primer sequences for each gene evaluated are listed in Supplementary Table 16. GAPDH was used as a loading control for morpholino validation. Samples were run on 2% agarose gels and imaged on a Fusion FX Vilber Lourmat chemiluminescence imaging system.

### siRNA silencing of LASR complex members and select DSGs

Gene silencing of LASR complex members and select DSGs was performed using Silencer Select siRNAs (Life Technologies) at 30 nM (HDMB03) or 15 nM (MB3W1) concentrations. A non-silencing scramble siRNA was utilized as a negative control. siRNA sequences for all genes are provided in Supplementary Table 17. Gene silencing was validated by immunoblotting. A list of the antibodies used for validation studies is provided in Supplementary Table 18.

### ChIP-qPCR

HDMB03 cells were seeded at 2 × 10^5^ cells per well under stem cell conditions and cultured for 72 h. Cells were then fixed and quenched using 1% paraformaldehyde and 0.125 M glycine, respectively. Lysates were next sonicated to 100–500 base pair fragments followed by incubation overnight with primary antibodies (Supplementary Table 18) at 4 °C. Protein A Dynabeads (Invitrogen) were used to capture antibody/chromatin complexes for 4 h followed by washing, elution and reverse crosslinking. Chromatin was purified using the Qiagen PCR Purification Kit. Chromatin was amplified using primers designed to flank known OTX2 binding sequences (Supplementary Table 16) and GoTaq qPCR master mix (Promega). Fold enrichment of OTX2 binding was calculated and graphed with Prism.

### Overexpression of wild-type OTX2 and OTX2 lacking its homeodomain

Wild-type and homeodomain-deleted human *OTX2* sequences cloned into the plasmid pcDNA3.1^+^ were purchased from GenScript. The coding sequences were amplified by PCR, adding overlap sequences to the 5′ ends of primers to allow NEBuilder HiFi assembly (New England Biolabs) with NheI/ClaI-linearized pCAG-H2B-mAG-P2A-3XHA-TurboID^3^, yielding pCAG-H2B-mAG-P2A-3XHA-hOTX2 and pCAG-H2B-mAG-P2A-3XHA-hOTX2ΔHD. All cloning reagents were purchased from New England Biolabs. The sequences of the resultant plasmids were verified by whole-plasmid sequencing (Eurofins Genomics).

### Pseudotime analysis of cells from the developing human cerebellum

Single-nucleus RNA-seq data from the developing human cerebellum were obtained and processed as previously described^31^. Briefly, sample fastqs were aligned to the human reference genome hg19 and the resultant count data were interpreted in Seurat (version 4.1.0)^66^ using the R environment (version 4.1.3) in order to perform quality control, normalization by SCTransform^67^, dimensionality reduction (uniform manifold approximation and projection), clustering (shared nearest neighbour and Louvain modularity optimization) and cell type identification. Trajectory inference within the developing human glutamatergic cells was also performed as previously described^3^. Briefly, Slingshot (version 1.6.1)^68^ was used to find the expected granule neuron and UBC lineages and TradeSeq (version 1.2.01)^69^ was used to statistically correlate gene expression with lineage pseudotime values. The resulting significant lineage-associated genes were then used in downstream analyses.

### Protein–protein interaction networks

Protein–protein interactions (PPIs) within both the OTX2-regulated DSGs list and the OTX2 TurboID hits list were determined by querying the STRING database (version 11.5)^70^. Briefly, gene lists were inputted and the resulting networks of PPIs were further filtered for only known physical interactions (‘Experiments = T; Databases = T’) with confidence scores of at least 0.150 (low confidence) or 0.400 (medium confidence), where indicated. This list of known PPIs was visualized using Cytoscape (version 3.8.0)^71^. Nodes were coloured by relevant enriched pathways in the queried lists using g:Profiler^72^ with the Gene Ontology Biological Processes (GO:BP), KEGG, Reactome and WikiPathways databases and an FDR threshold of 0.1. A full list of enriched pathways is provided in Supplementary Table 2. Nodes were manually rearranged to prevent node overlap; thus, the edge length is not representative of any value.

### Immunoblotting

Protein quantities (10–25 μg) were loaded onto 10–12% Tris-glycine gels and resolved by sodium dodecyl sulfate polyacrylamide gel electrophoresis followed by transferral to nitrocellulose membranes, blocking with 5% milk and incubation overnight with select primary antibodies (details provided in Supplementary Table 18). Secondary antibodies conjugated to horseradish peroxidase were added for 1 h at room temperature followed by detection using SuperSignal West Pico. A Fusion FX Vilber Lourmat chemiluminescence imaging system was used to capture the images.

### Immunofluorescence

HDMB03 cells were plated at 5 × 10^4^ cells per well in 24-well plates on glass coverslips that had been coated in poly-d-lysine (Thermo Fisher Scientific) for 10 min before being washed with molecular-grade H_2_O. For OTX2 silencing, HDMB03 cells were treated with scrambled RNA (negative control) or Silencer Select siRNA 9931 (Supplementary Table 17). HDMB03 cells were transfected with *OTX2*^WT^ or *OTX2*Δ^HD^ plasmid (the latter resulting in deletion of the DNA-binding homeodomain) 24 h after plating, as described. At 72 h after HDMB03 cells were plated, they were washed three times with phosphate-buffered saline (PBS) and fixed with 4% paraformaldehyde at room temperature for 30 min. Cells were then washed three times with PBS before permeabilization with 0.1% Triton X-100 in PBS for 20 min at room temperature. After washing three times with PBS, cells were blocked with 3% lamb serum/PBS for 1 h at room temperature, followed by replacement of the blocking buffer with 1:100 primary antibody (RBFOX2, HNRNPC or HNRNPM) (Supplementary Table 18) in 1% lamb serum/PBS. After 1 h incubation at room temperature, the cells were washed three times with PBS before incubating with either anti-rabbit (RBFOX2 and HNRNPM) or anti-mouse (HNRNPC) secondary antibody. For the *OTX2* silencing and *OTX2* deletion mutation experiments, Alexa Fluor 488 and Alexa Fluor 594 were used, respectively (Supplementary Table 18), at a dilution of 1:1,000 for 1 h at room temperature. Cells were then washed three times with PBS and once with H_2_O, followed by mounting with VECTASHIELD Antifade Mounting Medium with DAPI (Vector Laboratories). This was performed by placing a drop of mounting medium on a glass slide and flipping the coverslip with the cells onto the drop of mounting medium. Images were taken at 40× magnification using an EVOS M5000 Imaging System and the cytoplasmic area per cell was quantified using CellProfiler^73^.

### OTX2 TurboID and mass spectrometry

TurboID experiments were performed as previously described by Cho et al.^74^. NEBuilder HiFi DNA Assembly (New England Biolabs) was used to introduce the human *OTX2* sequence into the plasmid pCAG-H2B-mAG-P2A-3xHA-TurboID^3^. The vector was linearized with KpnI and ClaI and assembled with PCR-amplified 3xHA-TurboID (FW_KpnI_Turbo and RV_Turbo_GGSGG primers) and PCR-amplified hOTX2 (using reverse-transcribed RNA (Norgen) recovered from HDMB03 cells and RNEB_ClaI_N-Otx2 and FNEB_Link_N-OTX2 primers) to generate pCAG-H2B-mAG-P2A-3xHA-TurboID-OTX2. PCR-amplified sequences in the final plasmid were verified by Sanger sequencing. The primers used were as follows: FW_KpnI_Turbo (5′-AGAACCCTGGACCTGGTACCATGTACCCGTATGATGTTCCGG-3′); RV_Turbo_GGSGG (5′-GCCTCCAGATCCGCCCTTTTCGGCAGACCGCAGACTG-3′); RNEB_ClaI_N-OTX2 (5′-CGAGCTCTAGATCATCGATTTACAAAACCTGGAATTTCCACGAGGATGTCTG-3′); and FNEB_Link_N-OTX2 (5′-GGCGGATCTGGAGGCATGATGTCTTATCTTAAGCAACCGCCTTAC-3′).

For the TurboID transfection studies, 5 × 10^5^ HDMB03 cells per well were seeded into 6-well ultra-low attachment plates and transfected using Lipofectamine 3000 (Thermo Fisher Scientific) with 12 μg plasmid, as previously described by Hendrikse et al.^3^. Tumourspheres were cultured for 72 h in StemPro Medium (Life Technologies) and then treated with 500 μM biotin-d (B4639; Sigma–Aldrich) for 15 min. Tumourspheres were then washed in 1× PBS and lysed with 1 ml 1× RIPA (20 mM Tris-HCl (pH 7.5), 150 mM NaCl, 1 mM EDTA-Na_2_, 1 mM EGTA, 1% NP-40, 1% sodium deoxycholate, 2.5 mM sodium pyrophosphate, 1 mM β-glycerophosphate, 1 mM Na_3_VO_4_ and 1× Halt Protease and Phosphatase Inhibitor (Thermo Fisher Scientific)) for 10 min^3^. Samples were then sonicated over 2 × 10 s pulses at 30% load to shear DNA (an FB50 sonicator with microprobe; Thermo Fisher Scientific) and clarified by centrifugation at 16,000*g* and 4 °C for 10 min. Supernatants were quantified using the Bradford protein assay. Streptavidin–sepharose bead slurries (50 µl aliquots) were washed once in 1 ml 1× RIPA. Washed beads were resuspended in 1 ml 1× RIPA containing 2 mg protein supernatant and rotated at 4 °C overnight. The next day, samples were centrifuged for 5 min at 1,000*g* and bead pellets were washed twice in 1 ml 2% sodium dodecyl sulfate solution (in double-distilled water) and then three times (8 min each time) in 1 ml Wash Buffer (50 mM Tris-HCl (pH 7.4) and 8 M urea) at room temperature. Samples were resuspended in Storage Buffer (285 μl of ammonium bicarbonate (50 mM) and 15 μl of 1 mM biotin) and stored on ice.

An Orbitrap Exploris 480 instrument (Thermo Fisher Scientific) was used to obtain mass spectrometry data, as described by Hendrikse et al.^3^ Briefly, all mass spectrometry raw files were processed with Proteome Discoverer (version 2.20.388) at the Manitoba Centre for Proteomics and Systems Biology. The files were searched for tryptic peptides against the human UniProt protein database (December 2020) using SEQUEST with standard Orbitrap settings, as previously described^3^. In addition, up to two missed cleavages were permitted, with a parent and fragment mass tolerance of 0.02 Da and 15 ppm. A fixed modification of cysteine carbamidomethylation was applied. Also, variable modifications including deamidation (at Asn and Gln), amino-terminal acetylation, oxidation (at Met and Trp), phosphorylation (at Ser, Thr and Tyr), ubiquitylation (at Lys), double oxidation (at Met and Trp) and biotinylation (at Lys) were permitted. The results were filtered by 1% FDRs at both the peptide and protein levels. SAINTexpress (version 1.0.0)^47,48^ was used to calculate the probability of each potential proximal protein interaction from background contaminants using default parameters (*n* = 4 OTX2 biological replicates and *n* = 3 control biological replicates). A complete list of OTX2-interacting proteins is provided in Supplementary Table 1.

### Co-IP

HDMB03 MB cells were grown as tumourspheres for 3 d, dissociated and lysed with immunoprecipitation lysis buffer (25 mM Tris (pH 7.4), 150 mM NaCl, 1% Triton X-100 and 5 mM EDTA) plus 1× protease inhibitor complex. Samples were then pre-cleared for 2 h and rotated at 4 °C. Immunoprecipitation was carried out overnight with 2 μg OTX2 antibody or immunoglobulin G. For co-IP on HDMB03 cells overexpressing wild-type OTX2 or OTX2 lacking its DNA-binding homeodomain, immunoprecipitation was carried out with a haemagglutinin antibody. Magnetic beads were blocked with 1% bovine serum albumin solution for 1 h, washed and then added to the protein/antibody complexes for 3 h with rotation at 4 °C. Beads were then washed five times with immunoprecipitation lysis buffer. Complexes were eluted by boiling for 5 min in 1× sodium dodecyl sulfate sample buffer. Interaction with LASR complex members (RBFOX2, ILF2, ILF3, MATR3, DDX5, HNRNPM, HNRNPC and HNRNPH) was then determined by immunoblotting. The antibody conditions are described in Supplementary Table 18.

### Subcellular fractionation

Subcellular fractionation was performed using previously established protocols^17,30^. HDMB03 cells were lysed in cytoplasmic lysis buffer (CLB; 340 mM sucrose, 10 mM Tris-HCl (pH 7.9), 0.1 mM EDTA, 3 mM CaCl_2_, 1 mM dithiothreitol (DTT), 2 mM MgCl_2_, 0.5% NP-40 and protease and phosphatase inhibitors) on ice for 10 min and the cytoplasmic fraction was removed following centrifugation at 3,500*g* for 15 min. Cellular pellets were then washed with CLB wash buffer (CLB lacking NP-40) followed by centrifugation at 800*g* for 3 min. Soluble nuclear fractions were removed by lysing the cells for 5 min on ice with nuclear lysis buffer (10% glycerol, 3 mM EDTA, 20 mM HEPES (pH 7.9), 150 mM KOAc, 1.5 mM MgCl_2_, 1 mM DTT, 0.1% NP-40 and protease and phosphatase inhibitors) followed by centrifugation at 15,000*g* for 30 min. The RNA fraction was then isolated by lysing cells with nuclease incubation buffer (10% glycerol, 150 mM HEPES (pH 7.9), 1.5 mM MgCl_2_, 1 mM DTT, 150 mM KOAc and protease and phosphatase inhibitors) plus 10 mg ml^−1^ RNase, whereas the DNA fraction was isolated by lysing cells with nuclease incubation buffer plus DNAse I (1 U) for 30 min at 37 °C. RNA and DNA fractions were then recovered by centrifugation at 20,000*g* for 30 min. Samples were analysed by sodium dodecyl sulfate polyacrylamide gel electrophoresis with the antibodies described in Supplementary Table 18.

### Intracerebellar transplantation and drug treatment

For the in vivo morpholino studies, HDMB03 and MB3W1 cells were treated with 1 or 2.5 μM control or *PPHLN1*-Mo for 5 d in culture. Cells were dissociated and 1 × 10^5^ (HDMB03) or 2 × 10^5^ (MB3W1) cells were injected into the cerebellums of 7- to 9-week-old male NOD-SCID mice. NOD-SCID mice were housed in individually ventilated cages (Tecniplast). Irradiated feed was used and bedding was sterilized by steam autoclave. The room ambient temperature was 21–23 °C with a relative humidity target of 50% (the range was 30–60%). Beginning at 06:00 am, the light cycle was 12 h on and 12 h off. The endpoint was defined as a 20% weight reduction from peak body weight and/or significant ataxia and ruffled fur. MRI was performed on a cryogen-free FlexiScan 7T system (MR Solutions). For tumour size calculations from MRI images, the ImageJ freehand tool was used to determine the volume from 18 serial sections (300 µm thickness) from each sample. The sum of all of the slices was then used to calculate an overall tumour volume, as previously described^55^. At the endpoint, tumours were prepared for immunohistochemistry using a mitochondria-specific antibody to detect human cells as well as SOX2 using methods previously described^15^. The primary and secondary antibody sources and concentrations are listed in Supplementary Table 18.

### Digital spatial profiling on MB xenograft tumour samples

One Ctrl-Mo and one *PPHLN1*-Mo formalin-fixed, paraffin-embedded xenograft tumour sample were processed following the GeoMx DSP Slide Preparation User Manual (MAN-10087-04). Briefly, slides were baked at 60 °C for at least 1 h, deparaffinized using CitriSolv d-limonene and then rehydrated. Antigen retrieval was performed using 1× citrate buffer at a pH of 6.0 in a pressure cooker for 15 min at 100 °C on high pressure. Slides were blocked using NanoString blocking buffer W for 1 h. Slides were then incubated overnight at 4 °C with the following ultraviolet-photocleavable, barcode-conjugated antibody panels against a total of 43 targets and six control targets from NanoString Technologies: GeoMx Neural Cell Profiling Panel Human Protein Core for nCounter; GeoMx Cell Death Panel Human Protein Module for nCounter; GeoMx PI3K/AKT Signaling Panel Human Protein Module for nCounter; and GeoMx MAPK Signaling Panel Human Protein Module for nCounter. At the same time, the samples were incubated with morphology antibodies consisting of a 1:100 dilution of Ki67–Alexa Fluor 647 (12075S; Cell Signaling Technology) and a 1:500 dilution of MAP2–Alexa Fluor 532 (NBP1-92711AF532; Novus Biologicals). The slides were washed and stained with SYTO 13 (S7575; Thermo Fisher Scientific) for 15 min before loading onto a GeoMx DSP microscope (NanoString Technologies). Fluorescence images were scanned at 20× and regions of interest (ROIs) along the tumour border and in the tumour core were selected. Oligos from antibodies were cleaved and collected into 96-well plates. Oligos were dried down completely by incubating overnight at room temperature with a permeable plate seal and then rehydrated and hybridized with NanoString HybCode barcodes. Samples were hybridized overnight for at least 16 h in a thermal cycler at 67 °C with a heated lid at 72 °C. The resulting oligos hybridized to barcode tags were detected and counted using an nCounter Prep Station and Digital Analyzer (NanoString Technologies) the following day. The digital counts of each antibody for each ROI were generated for data analyses and analysed using NanoString GeoMx software. The software has built-in quality control analysis and three ROIs that did not pass quality control metrics due to high binding density were excluded from downstream analysis. The data were normalized by scaling to the negative control immunoglobulin G probes, constituting a signal-to-noise ratio. Counts were further scaled to the number of nuclei per ROI and the normalized counts from the Ctrl-Mo and *PPHLN1*-Mo samples were calculated.

### Statistics and reproducibility

Prism 8.0 software (GraphPad) was used for all of the statistical analyses. No data were excluded from the analyses. NOD-SCID mice for the animal studies were randomly assigned into treatment groups; however, the remaining experiments were not randomized. Blinding was performed during MRI imaging and tissue preparation for immunohistochemistry. For all of the other experiments, the investigators were not blinded. No statistical methods were used to predetermine sample size, but our sample sizes are similar to those reported in previous publications^15,55^. Brown–Forsythe tests were performed to assess the homogeneity of variances for cell culture-based studies. The statistical tests used for all of the experiments are noted in the figure captions.

### Reporting summary

Further information on research design is available in the Nature Portfolio Reporting Summary linked to this article.

## Online content

Any methods, additional references, Nature Portfolio reporting summaries, source data, extended data, supplementary information, acknowledgements, peer review information; details of author contributions and competing interests; and statements of data and code availability are available at 10.1038/s41556-024-01460-5.

### Supplementary information

Reporting Summary

Supplementary Information Supplementary Tables 1–18.

### Source data

Source Data Figs. 2–6 and Extended Data Figs. 1–3, 6, 7, 9 and 10 Statistical source data.

Source Data Figs. 1–3, 5 and 6 and Extended Data Figs. 1, 3, 6, 7, 9 and 10 Unprocessed blots and gels.

## Data Availability

Sequencing data that support the findings of this study have been deposited in the GEO under accession code GSE222699 (*PPHLN1*-Mo RNA-seq data). TurboID data have been deposited in the ProteomeXchange Consortium via the PRIDE partner repository with the dataset identifier PXD052687. Previously published datasets that were re-analysed here are available as follows: HDMB03 and MB3W1 bulk RNA-seq data in siCtrl and siOTX2 tumourspheres are available from the GEO via accession code GSE189238; bulk RNA-seq data from retinoblastoma and normal retina samples are available from the GEO via accession codes GSE196420 (ref. ^32^) and GSE99248 (ref. ^33^), respectively; ChIP-seq data are available from the GEO via accession code GSE98354; bulk RNA-seq data of patient MB samples are available from the European Genome-Phenome Archive via accession codes EGAS00001005826 (ref. ^3^), EGAD00001006305 (ref. ^75^), EGAD00001004435 (ref. ^76^), EGAD00001005131 (ref. ^76^) and EGAD00001004958 (ref. ^52^); and single-nucleus RNA-seq data from the developing human cerebellum are available from the Human Cell Atlas (https://explore.data.humancellatlas.org/projects/85a9263b-0887-48ed-ab1a-ddfa773727b6), UCSC Cell Browser (https://cbl-dev.cells.ucsc.edu) or Database of Genotypes and Phenotypes (accession code phs001908.v2.p1). Data were also obtained through correspondence (Kimberly.Aldinger@seattlechildrens.org) with Aldinger et al.^31^. Developing human cerebellum bulk RNA-seq data were obtained through correspondence (parthiv.haldipur@seattlechildrens.org) with Haldipur et al.^6^ and are available through the Database of Genotypes and Phenotypes (accession code phs001908.v2.p1). Source data are provided with this paper. All other data supporting the findings of this study are available from the corresponding author upon request.
