# Supplementary material for: A group 3 medulloblastoma stem cell program is maintained by OTX2-mediated alternative splicing
Source: Nat Cell Biol. 2024 Jul 18;26(8):1233–46. doi: 10.1038/s41556-024-01460-5 (PMC11321995; doi:10.1038/s41556-024-01460-5)
Supplement: Supplementary file 1 — Reporting Summary [file 41556_2024_1460_MOESM1_ESM.pdf]

nature portfolio

Corresponding author(s): Tamra Werbowetski-Ogilvie

Last updated by author(s): May 24, 2024

Reporting Summary

Nature Portfolio wishes to improve the reproducibility of the work that we publish. This form provides structure for consistency and transparency in reporting. For further information on Nature Portfolio policies, see our Editorial Policies and the Editorial Policy Checklist.

Statistics

For all statistical analyses, confirm that the following items are present in the figure legend, table legend, main text, or Methods section.

n/a	Confirmed	
☐	☒	The exact sample size (*n*) for each experimental group/condition, given as a discrete number and unit of measurement
☐	☒	A statement on whether measurements were taken from distinct samples or whether the same sample was measured repeatedly
☐	☒	The statistical test(s) used AND whether they are one- or two-sided<br>*Only common tests should be described solely by name; describe more complex techniques in the Methods section.*
☐	☒	A description of all covariates tested
☐	☒	A description of any assumptions or corrections, such as tests of normality and adjustment for multiple comparisons
☐	☒	A full description of the statistical parameters including central tendency (e.g. means) or other basic estimates (e.g. regression coefficient) AND variation (e.g. standard deviation) or associated estimates of uncertainty (e.g. confidence intervals)
☐	☒	For null hypothesis testing, the test statistic (e.g. *F*, *t*, *r*) with confidence intervals, effect sizes, degrees of freedom and *P* value noted<br>*Give P values as exact values whenever suitable.*
☒	☐	For Bayesian analysis, information on the choice of priors and Markov chain Monte Carlo settings
☒	☐	For hierarchical and complex designs, identification of the appropriate level for tests and full reporting of outcomes
☒	☐	Estimates of effect sizes (e.g. Cohen's *d*, Pearson's *r*), indicating how they were calculated

Our web collection on statistics for biologists contains articles on many of the points above.

Software and code

Policy information about availability of computer code

Data collection	Bulk RNA sequencing was performed using the Illumina HiSeq 2000 or 2500 platform and aligned to the hg19 reference using STAR (v2.5.2b) software. Mass spectrometric data were obtained with an Orbitrap Exploris 480 instrument, raw output files were processed with Proteome Discoverer (v2.20.388) against the human Uniprot protein database (Dec, 2020) using SEQUEST.
Data analysis	Bulk RNA sequencing data were analyzed using R (v4.0.2) implementing the following published algorithms which are further described and cited in the methods section: STAR aligner (version 2.5.2b), DEseq2 (DESeq2_1.26.0), HTSeq (v.0.6.1p1), rMATS (v3.0.9), ggsashimi.py script (https://github.com/guigolab/ggsashimi) and survival (v3.2-11).<br>Enrichment analysis was performed using the ClusterProfiler tool (4.0)<br>Single nucleus RNA sequencing data were analyzed using R (v4.0.2) implementing the following published algorithms which are further described and cited in the methods section: Seurat (v.4.1.0), Slingshot (v1.6.1), TradeSeq (v1.2.01), ComplexHeatmap (v.2.0.0), SingleCellExperiment (v1.6.0).<br>For ChIP-seq:: bowtie2 2.2.4, MACS2 v2.1.0.20140616

For manuscripts utilizing custom algorithms or software that are central to the research but not yet described in published literature, software must be made available to editors and reviewers. We strongly encourage code deposition in a community repository (e.g. GitHub). See the Nature Portfolio guidelines for submitting code & software for further information.

Data

Policy information about availability of data

All manuscripts must include a data availability statement. This statement should provide the following information, where applicable:

- Accession codes, unique identifiers, or web links for publicly available datasets
- A description of any restrictions on data availability
- For clinical datasets or third party data, please ensure that the statement adheres to our policy

Sequencing data that support the findings of this study have been deposited in the Gene Expression Omnibus (GEO) under the GEO accession number GSE222699 (PPHLN1-Mo RNA-seq data). TurboID data have been deposited to the ProteomeXchange Consortium via the PRIDE partner repository with the dataset identifier PXD052687.

Previously published data sets that were re-analyzed here are available as follows:
HDMB03 and MB3W1 bulk RNA sequencing data in siCTRL and siOTX2 tumorpsheres can be accessed from GEO under GSE189238.
Bulk RNA-seq data from retinoblastoma and normal retina samples were accessed from GEO under the accession codes GSE19642074 and GSE9924875 respectively.
ChIP-seq data can be accessed from GEO under GSE98354.
Bulk RNA-seq data of patient medulloblastoma samples was accessed from EGAS000010058265, EGAD0000100630585, EGAD000010044353, EGAD000010051313, and EGAD0000100495859.
Single-nucleus RNA-seq data from the developing human cerebellum are available through the Human Cell Atlas [https://www.covid19cellatlas.org/aldinger20], the UCSC Cell Browser [https://cbl-dev.cells.ucsc.edu], or from Database of Genotypes and Phenotypes (dbGaP) (accession number: phs001908.v2.p1). Data were also obtained through correspondence with Aldinger et al.33.
Developing human cerebellum bulk RNA-seq data were obtained through correspondence with Haldipur et al.,8 and are also available through dbGaP (accession number: phs001908.v2.p1).

All other data supporting the findings of this study are available from the corresponding author upon request.

Research involving human participants, their data, or biological material

Policy information about studies with human participants or human data. See also policy information about sex, gender (identity/presentation), and sexual orientation and race, ethnicity and racism.

Reporting on sex and gender	NA
Reporting on race, ethnicity, or other socially relevant groupings	NA
Population characteristics	NA
Recruitment	NA
Ethics oversight	NA

Note that full information on the approval of the study protocol must also be provided in the manuscript.

Field-specific reporting

Please select the one below that is the best fit for your research. If you are not sure, read the appropriate sections before making your selection.

☒ Life sciences ☐ Behavioural & social sciences ☐ Ecological, evolutionary & environmental sciences

For a reference copy of the document with all sections, see nature.com/documents/nr-reporting-summary-flat.pdf

Life sciences study design

All studies must disclose on these points even when the disclosure is negative.

Sample size	No statistical methods were used to pre-determine sample size, but our sample sizes are similar to those reported in previous publications (Zagozewski et al., 2020; Zagozewski and Borlase et al., 2022). For in vivo studies, sample size was determined based on availability of animals. For in vitro studies, all experiments were performed on a minimum of 3 independent biological replicates for each cell line unless otherwise

states. For patient sample transcriptome and proteome studies, sample size was based on the number of samples already present in publicly available datasets or made available to the lab through collaboration.

Data exclusions

No data exclusions were considered

Replication

For vitro/molecular experiments (ie. tumorsphere assays, RT-PCR, RNA sequencing, immunoblot), studies were carried out in 3 or greater independent biological replicates for each cell line unless otherwise indicated. Slides from multiple control and treatment group tumor sections were stained for IHC and representative images shown. In vivo analysis of PPHLN1-Mo treated xenografts was performed on 2-3 independent sets of NOD-SCID mice per cell line. Attempts at replication were successful.

Randomization

The experiments were not randomized.

Blinding

Blinding was performed during MRI imaging and tissue preparation for IHC. For all cell culture and molecular experiments, measurements were performed by 2 independent observers to minimize bias and ensure reproducibility.

Reporting for specific materials, systems and methods

We require information from authors about some types of materials, experimental systems and methods used in many studies. Here, indicate whether each material, system or method listed is relevant to your study. If you are not sure if a list item applies to your research, read the appropriate section before selecting a response.

Materials & experimental systems

n/a	Involved in the study	
☐	☒	Antibodies
☐	☒	Eukaryotic cell lines
☒	☐	Palaeontology and archaeology
☐	☒	Animals and other organisms
☒	☐	Clinical data
☒	☐	Dual use research of concern
☒	☐	Plants

Methods

n/a	Involved in the study	
☐	☒	ChIP-seq
☐	☒	Flow cytometry
☐	☒	MRI-based neuroimaging

Antibodies

Antibodies used

OTX2, immunoblotting, Abcam, ab21990, 1/500
OTX2, ChIP-sequencing, Abcam, ab21990, 10 ug
OTX2, immunoprecipitation, Proteintech, 13497-1-AP, 2 μg
Beta actin, immunoblotting, Sigma-Aldrich, A2228, 1/1000
GAPDH, immunoblotting, Santa Cruz Biotechnology, sc-47724, 1/2000
p-S6, immunoblotting, Cell Signaling Technology, 2211, 1/1000
p-4E-BP1, immunoblotting, Cell Signaling Technology, 9451, 1/1000
Total S6, immunoblotting, Cell Signaling Technology, 2317, 1/1000
Total 4E-BP1, immunoblotting, Cell Signaling Technology, 9644, 1/2000
DDX5, immunoblotting, Santa Cruz (A-5, sc-166167), 1/800
HA, immunoblotting and co-IP, Cell Signaling Technology, 2367S, 1/2000
HA, immunoblotting and co-IP, Cell Signaling Technology, 3724S, 1/2000
hnRNP M, immunoblotting, Cell Signaling Technology, 28699S, 1/500
hnRNP C1/C2, immunoblotting, Santa Cruz Biotechnology, sc-32308, 1/200
hnRNP F/H, immunoblotting, Santa Cruz Biotechnology, sc-32310, 1/200
MADD, immunoblotting, Abcam, ab134117, 1/1000
Matrin-3, immunoblotting, Bethyl A300-591A-M, 1/1000
ILF2, immunoblotting, Santa Cruz Biotechnology, sc-365283, 1/500
ILF3, immunoblotting, Santa Cruz Biotechnology, sc-377406, 1/200
PPHLN1, immunoblotting, Thermo Fisher, PA5-58584, 1/1000
RBFOX2 (rabbit), immunoblotting, Bethyl, A300-864A-M,1/750
RBFOX2 (mouse), immunoblotting, Thermo Fisher, MA5-38623, 1/500
Normal rabbit IgG, immunoprecipitation, Cell Signaling Technology, 2729, 2 μg
SOX2, IHC, Cell Signaling Technology, 3579, 1/100
Mitochondria, IHC, Abcam, ab92824, 1/500
Annexin V, Flow cytometry, BD Biosciences, 561012, as per manufacturer's guidelines
Biotin-SP sheep anti-mouse IgG, IHC, Jackson ImmunoResearch, 515-065-003, 1/500
Biotin-SP goat anti-rabbit IgG, IHC, Jackson ImmunoResearch, 111-065-144, 1/500
Goat anti-mouse HRP, immunoblotting, Abcam, ab6789, 1/3000
Donkey anti-rabbit HRP, immunoblotting, Jackson Immunoresearch, 711-035-152, 1/5000

Validation

OTX2 (ab21990) manufacturer tested applications include ChIP, immunoblot.
Beta actin (A2228), GAPDH (sc-47724), hnRNP C1/C2 (sc-32308), hnRNP F/H (sc-32310), MADD (ab134117), Matrin-3 (A300-591A-M), ILF2 (sc-365283), ILF3 (sc-377406), PPHLN1 (PA5-58584), RBFOX2 (A300-864A-M), RBFOX2 (MA5-38623) have been testing in

immunoblotting by the manufacturers.
Mitochondria (ab92824) has been tested in IHC by the manufacturers.

OTX2 (13497-1-AP) antibody underwent KD/KO validation and was tested for IP with Y79 lysates by the manufacturers.
p-4E-BP1 (9451), Total 4E-BP1 (9644), p-S6 (2211), total S6 (2317), Sox2 (3579), hnRNP M (28699S), Caspase-3 (9664) and Normal rabbit IgG (2729) antibodies are validated according to Cell Signaling Technologies Hallmarks of Antibody Validation™ which for immunoblotting may include KD/KO validation, validation across several cell lines/tissues, etc and for IHC may include staining with the use of blocking peptides, staining on human cancer tissue arrays, etc.
Annexin V (561012) was titrated by investigators to attain the appropriate concentrations.
Antibodies were also chosen based on the literature and further validated in our study by immunoblotting or IF and appropriate gain/loss of function studies. Antibodies were all commercially available as stated above and additional validation statements are available on the company website.

Eukaryotic cell lines

Policy information about cell lines and Sex and Gender in Research

Cell line source(s)	HDMB03 was kindly provided by Dr. Till Milde (Milde T, et al., Journal of Neuro-oncology, 2012)<br>MB3W1 was kindly provided by Dr. Matthias Wölfl (Dietl S, et al., BMC cancer, 2016)
Authentication	All cell lines have been authenticated by STR profiling (ATCC)
Mycoplasma contamination	Cell lines were not tested for mycoplasma contamination
Commonly misidentified lines<br>(See ICLAC register)	No cell lines utilized in our study are listed in the ICLAC register

Animals and other research organisms

Policy information about studies involving animals; ARRIVE guidelines recommended for reporting animal research, and Sex and Gender in Research

Laboratory animals	NOD-SCID male mice aged 7-9 weeks were utilized for all in orthotopic xenograft in vivo analysis.
Wild animals	Our study did not utilize wild animals
Reporting on sex	Group 3 MB affects males at much higher incidence (2:1) than females and therefore only male mice were utilized in this study. In addition, likely due to this higher incidence in males, almost all Group 3 cell lines available for study are derived from male patients. All cell lines used in this study are derived from male patients.
Field-collected samples	Our study did not involve field-collected samples
Ethics oversight	All in vivo procedures were approved by the University of Manitoba Animal Care Committee (AUP-22-005).

Note that full information on the approval of the study protocol must also be provided in the manuscript.

Plants

Seed stocks	N/A
Novel plant genotypes	N/A
Authentication	N/A

ChIP-seq

Data deposition

☒ Confirm that both raw and final processed data have been deposited in a public database such as GEO.

☒ Confirm that you have deposited or provided access to graph files (e.g. BED files) for the called peaks.

Data access links<br>*May remain private before publication.*	https://www.ncbi.nlm.nih.gov/geo/query/acc.cgi?acc=GSE98354
Files in database submission	GSE98354_OTX2_peaks.xls.gz
Genome browser session<br>(e.g. UCSC)	*Provide a link to an anonymized genome browser session for "Initial submission" and "Revised version" documents only, to enable peer review. Write "no longer applicable" for "Final submission" documents.*

Methodology

Replicates	one replicate of each condition:<br>GSE98354 OTX2<br>GSE98354 Input
Sequencing depth	Paired end 150bp<br>OTX2: 181055070 total, 175761999 mapped<br>Input: 68475232 total, 66522931 mapped
Antibodies	OTX2, ChIP-sequencing, Abcam, ab21990, 10 ug
Peak calling parameters	callpeak --gsize=hs --keep-dup=1 --qval=0.05 --treatment=OTX2.bam --control=Input.bam --format=BAMPE --name=OTX2
Data quality	FASTQC analysis was performed, mapping statistics were determined
Software	ChIP-Sequencing were mapped to GRCh38 using bowtie2 2.2.4, peak calling was performed using MACS2 2.1.0.

Flow Cytometry

Plots

Confirm that:

☒ The axis labels state the marker and fluorochrome used (e.g. CD4-FITC).

☒ The axis scales are clearly visible. Include numbers along axes only for bottom left plot of group (a 'group' is an analysis of identical markers).

☒ All plots are contour plots with outliers or pseudocolor plots.

☒ A numerical value for number of cells or percentage (with statistics) is provided.

Methodology

Sample preparation	The Annexin V Apoptosis Detection Kit (Annexin V-PE) (BD Biosciences) was used to evaluate cell death as previously described (Liang et al., Cancer Research, 2018; Zagozewski et al., 2020). In both cases, tumorsphere populations were dissociated using Accutase into single cell suspensions for further analyses. An unstained suspension served as a negative control, and single-stained suspensions as positive controls. These various controls were used for compensation and gating for flow cytometry analyses. Samples were acquired by flow cytometry (Gallios, Beckman Coulter, Indianapolis, USA), and analyzed using Kaluza Analysis Software (Beckman Coulter).
Instrument	Gallios, Beckman Coulter, Indianapolis, USA
Software	Kaluza software (Beckman Coulter)
Cell population abundance	Cell sorting was not performed in these studies, only analytical studies by flow cytometry.
Gating strategy	For Annexin V, gates were set based on unstained controls. A plot depicting gating strategy is included in Supplementary Figure 10i. An initial broad FSC/SSC gate was placed around the entire population only eliminating debris that is under a FSC-A/SSC-A threshold of 50.

☒ Tick this box to confirm that a figure exemplifying the gating strategy is provided in the Supplementary Information.

Magnetic resonance imaging

Experimental design

Design type	Resting state
Design specifications	N/A
Behavioral performance measures	N/A

Acquisition

Imaging type(s)	Structural
Field strength	7 Tesla
Sequence & imaging parameters	Fast spin echo T1, FOV 30 x 30 mm, matrix 512x 256, TR= 1000ms, TE=11ms, FA=90, coronal slice, 0.3mm slice

Sequence & imaging parameters

thickness
Fast spin echo T2, FOV 30 x 30 mm, matrix 256x 245, TR= 5000ms, TE=45ms, FA=90, coronal slice, 0.3mm slice thickness

Area of acquisition

Whole brain

Diffusion MRI ☐ Used ☒ Not used

Preprocessing

Preprocessing software	N/A
Normalization	N/A
Normalization template	N/A
Noise and artifact removal	N/A
Volume censoring	N/A

Statistical modeling & inference

Model type and settings	N/A
Effect(s) tested	N/A

Specify type of analysis: ☒ Whole brain ☐ ROI-based ☐ Both

Statistic type for inference (See Eklund et al. 2016)	N/A
Correction	N/A

Models & analysis

n/a	Involved in the study
☒	☐ Functional and/or effective connectivity
☒	☐ Graph analysis
☒	☐ Multivariate modeling or predictive analysis
