# Supplementary material for: A group 3 medulloblastoma stem cell program is maintained by OTX2-mediated alternative splicing
Source: Nat Cell Biol. 2024 Jul 18;26(8):1233–46. doi: 10.1038/s41556-024-01460-5 (PMC11321995; doi:10.1038/s41556-024-01460-5)
Supplement: Supplementary file 4 — Unprocessed blots and gels. [file 41556_2024_1460_MOESM4_ESM.pdf]

Figure 1d

Figure 1d continued

Figure 1e

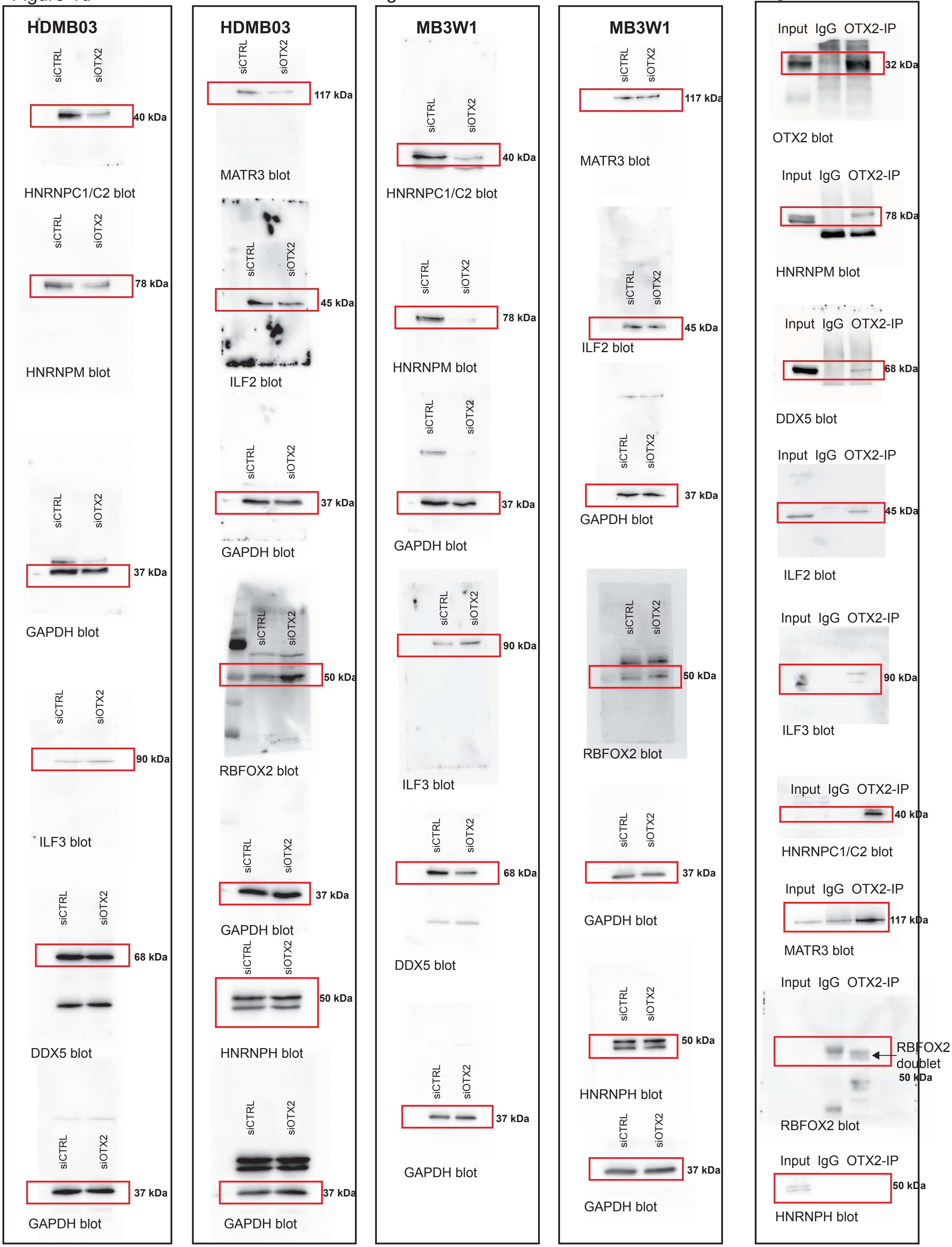

Figure 2a

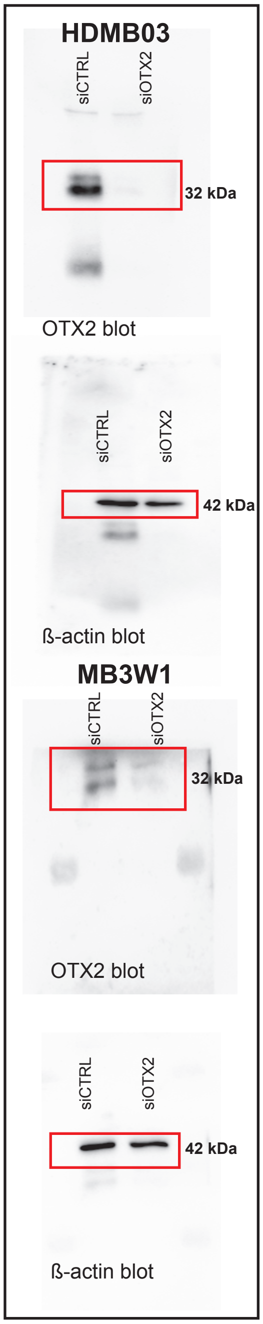
HDMB03
siCTRL
siOTX2
32 kDa
OTX2 blot
siCTRL
siOTX2
42 kDa
ß-actin blot
MB3W1
siCTRL
siOTX2
32 kDa
OTX2 blot
siCTRL
siOTX2
42 kDa
ß-actin blot

gels

Figure 3d

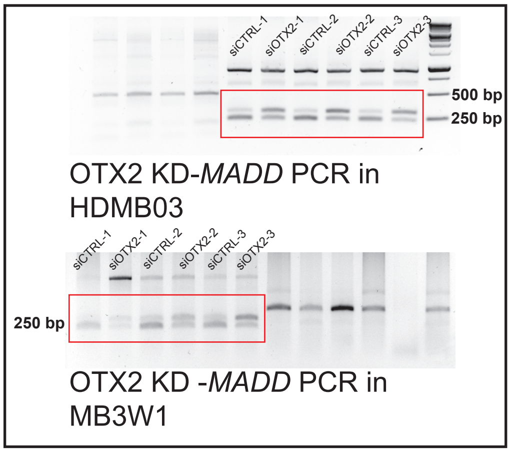

Figure 3g

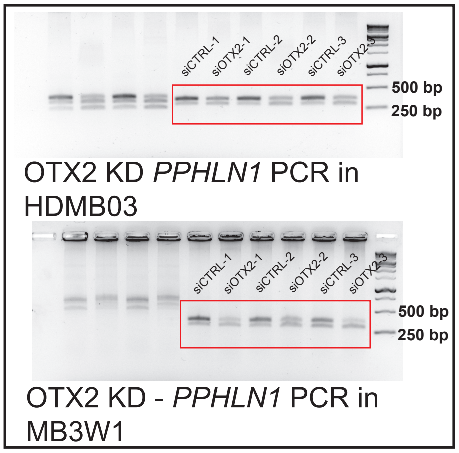

Figure 3k

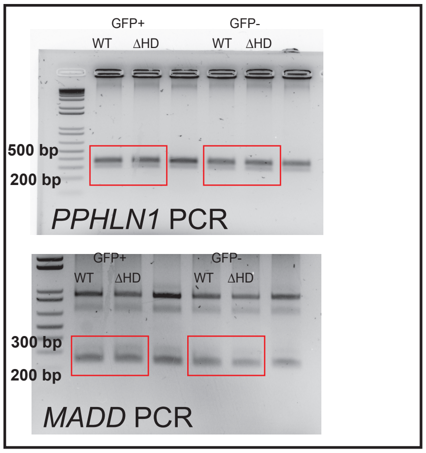

westerns

Figure 3j

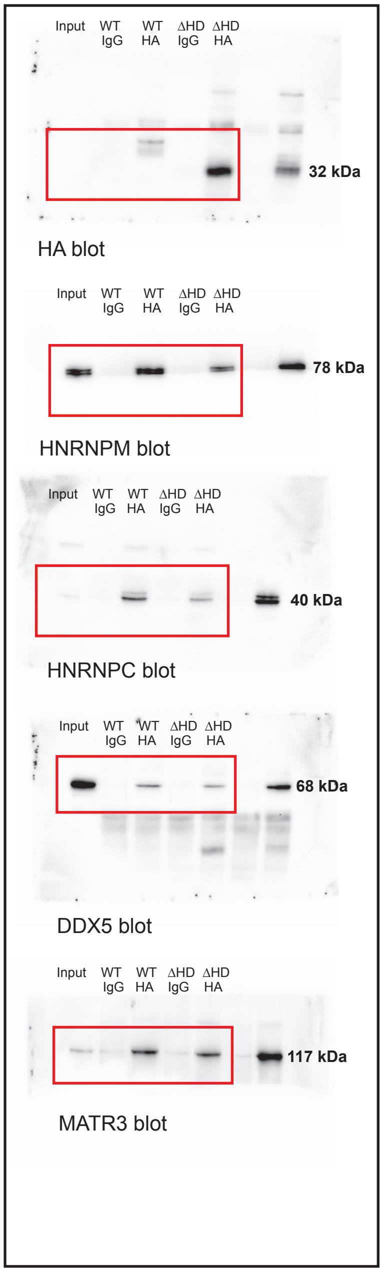

Figure 3l

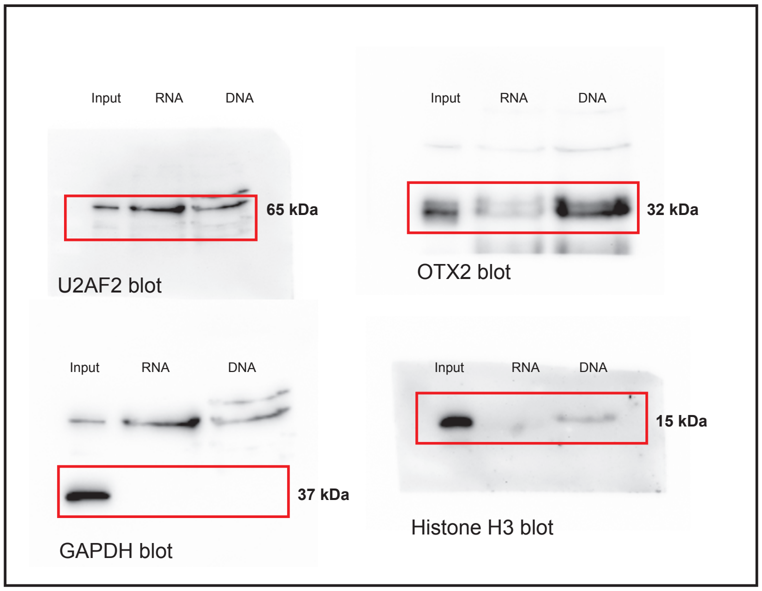

Figure 5b

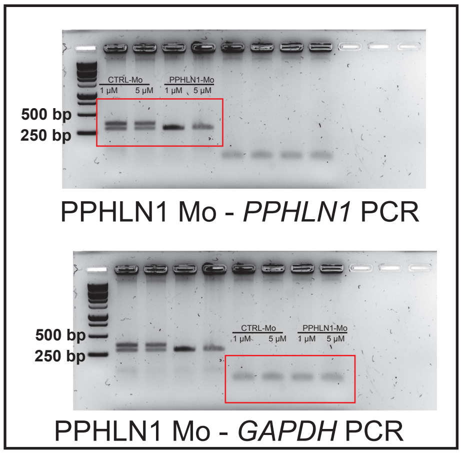

Figure 5c

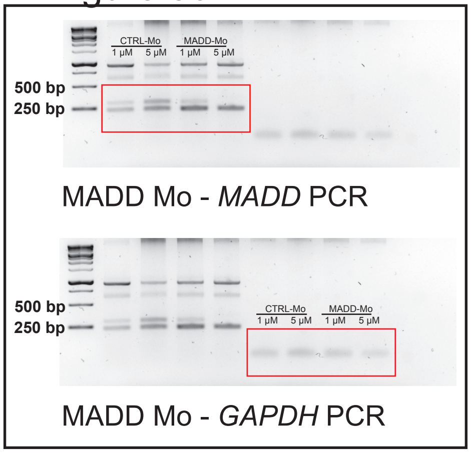

Figure 5e

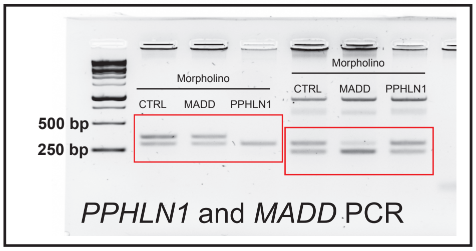

Figure 5n

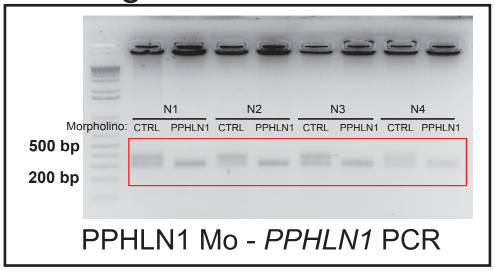

westerns

Figure 6a

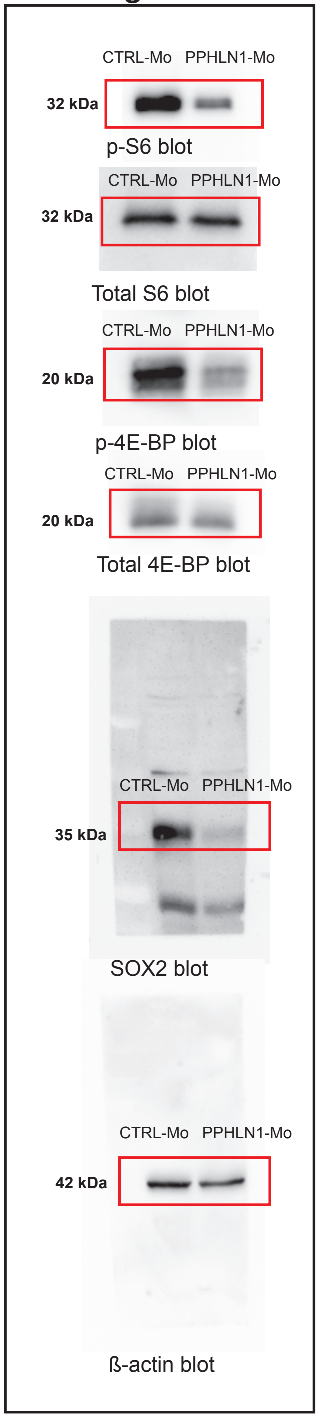

Figure 6b

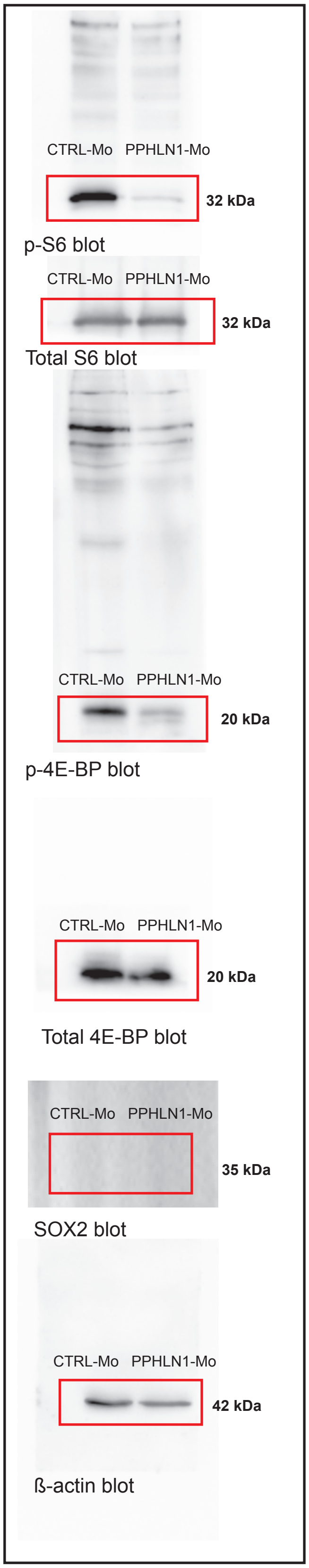

gels

Figure 6c

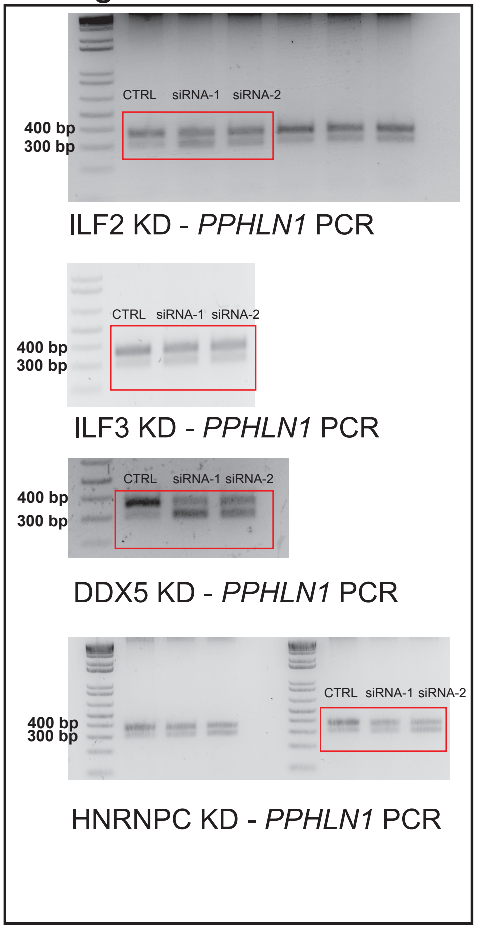

Figure 6d

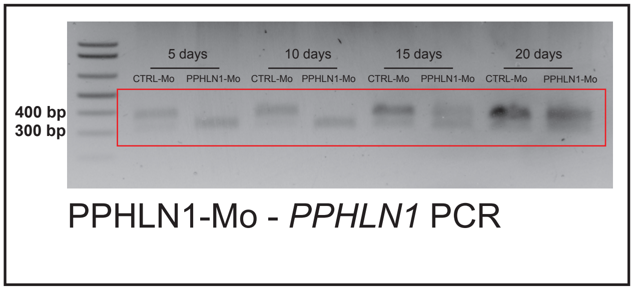

Extended Data Figure 1a

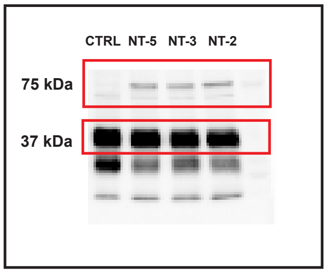

Extended Data Figure 1b

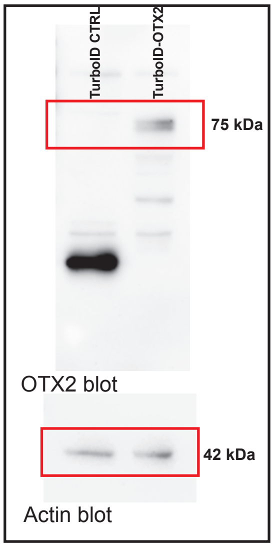

Extended Data Figure 3a

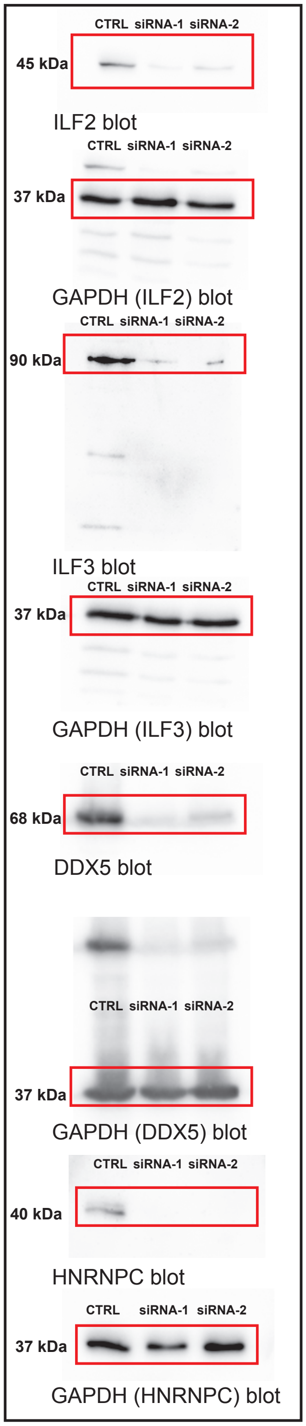

Extended Data Figure 3a continued

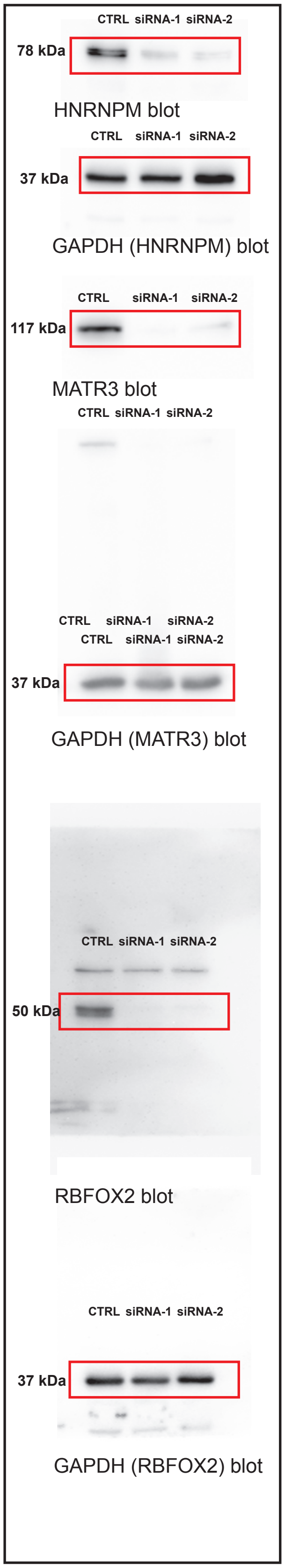

Extended Data Figure 3d

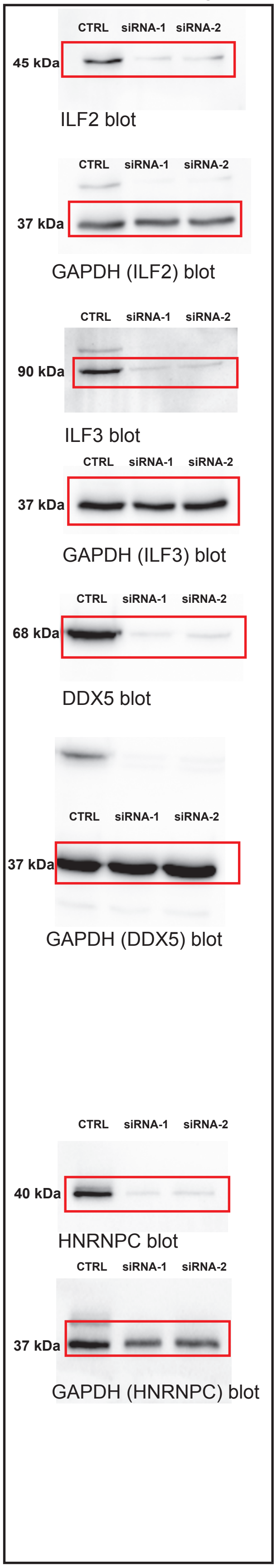

Extended Data Figure 6b

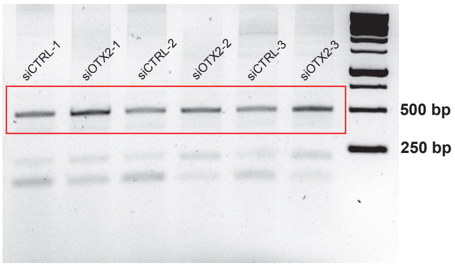

OTX2 KD - *MBD1* PCR in HDMB03

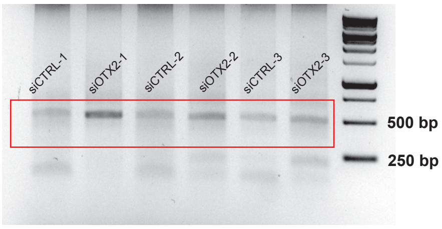

OTX2 KD - *MBD1* PCR in MB3W1

Extended Data Figure 6d

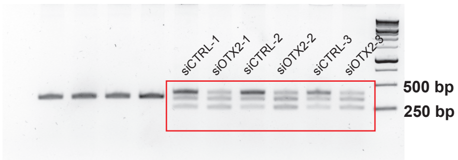

OTX2 KD - *PAPOLA* PCR in HDMB03

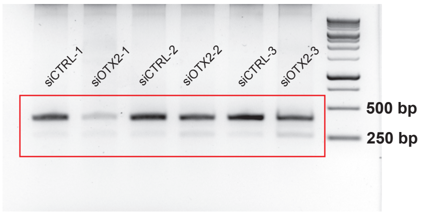

OTX2 KD - *PAPOLA* PCR in MB3W1

Extended Data Figure 7b

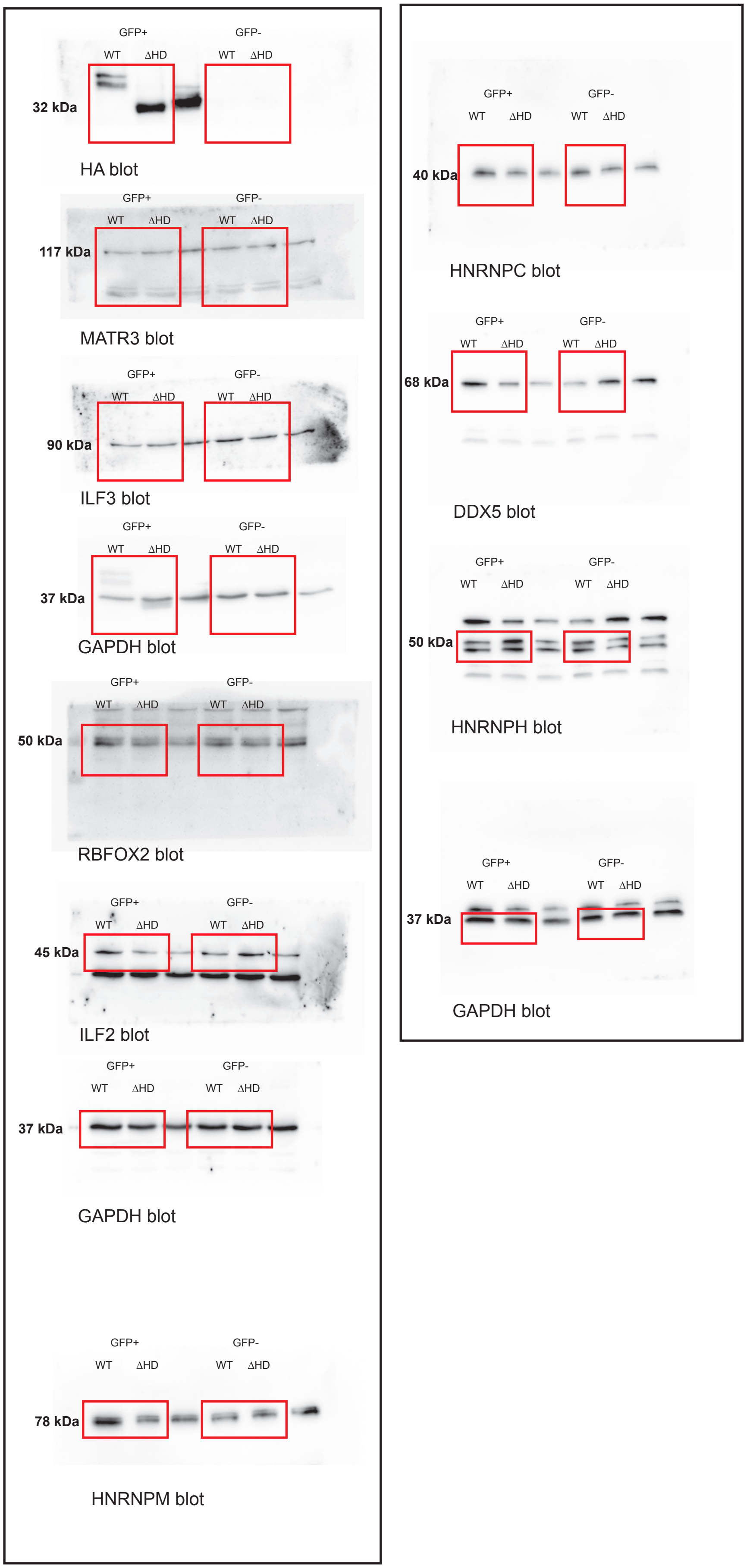

Extended Data Figure 9c

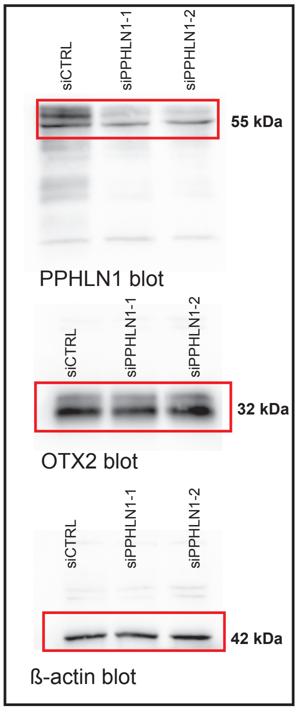

Extended Data Figure 9d

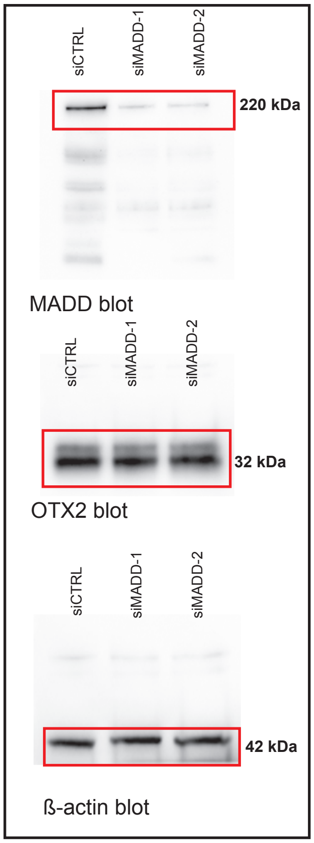

Extended Data Figure 9m

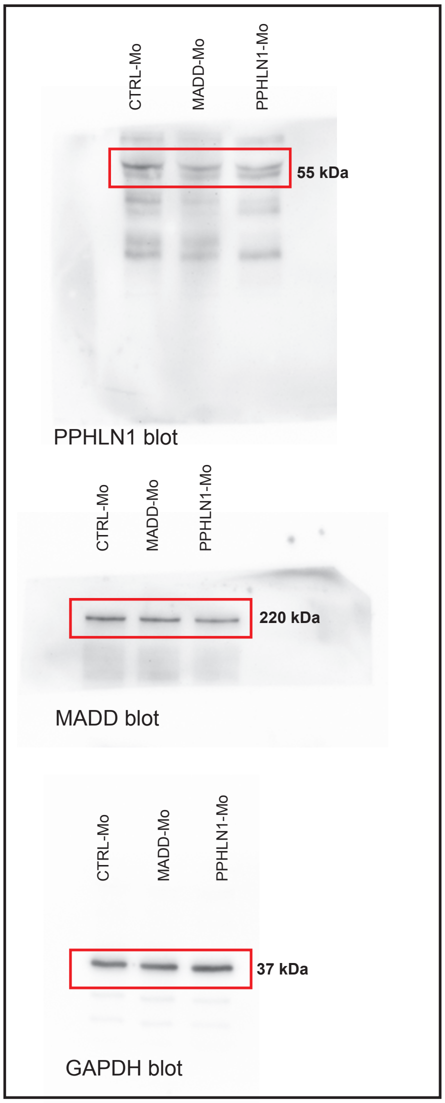

Extended Data Figure 10a

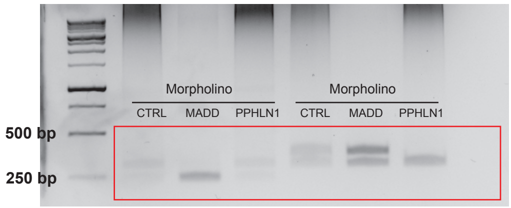

5 uM Mo treatment in MB3W1

Left - *MADD* PCR; Right *PPHLN1* PCR
